# Firearm Access and Gun Violence Exposure Among American Indian or Alaska Native and Black Adults

**DOI:** 10.1001/jamanetworkopen.2024.0073

**Published:** 2024-03-04

**Authors:** Michael D. Anestis, Jayna Moceri-Brooks, Devon Ziminski, R. Thurman Barnes, Daniel Semenza

**Affiliations:** 1 New Jersey Gun Violence Research Center, Rutgers, The State University of New Jersey, Piscataway; 2 School of Public Health, Rutgers, The State University of New Jersey, Piscataway; 3 Senator Walter Rand Institute for Public Affairs, Rutgers University–Camden, Cinnaminson, New Jersey; 4 Department of Sociology, Anthropology, and Criminal Justice, Rutgers University–Camden, Cinnaminson, New Jersey

## Abstract

**Importance:**

American Indian or Alaska Native and Black adults experience elevated rates of firearm injury and death, but both groups are severely underrepresented in research on firearm exposure and behaviors.

**Objective:**

To explore geodemographic differences in firearm behaviors and violence exposure among American Indian or Alaska Native and Black adults in the US.

**Design, Setting, and Participants:**

In this survey study, nationally representative samples of American Indian or Alaska Native and/or Black adults recruited from KnowledgePanel were surveyed cross-sectionally. Surveys were administered online between April 12 and May 4, 2023.

**Main Outcomes and Measures:**

Firearm access, storage, and carrying behaviors and lifetime firearm violence exposure were the primary outcomes. Demographic factors such as age, geographic location, and political affiliation were considered. Data were weighted to geodemographic distributions from the US Census Bureau’s 2022 Current Population Survey.

**Results:**

Of 3542 participants, 527 (14.9%) were American Indian or Alaska Native (280 [53.1%] female) and 3015 (85.1%) were Black (1646 [54.6%] female). Both groups exhibited high firearm access rates (American Indian or Alaska Native adults: 238 [45.4%; 95% CI, 39.4%-51.7%]; Black adults: 909 [30.4%; 95% CI, 28.0%-32.9%]), predominantly owning handguns for home protection. The groups demonstrated similar firearm storage patterns, and a substantial proportion endorsed always or almost always carrying firearms outside the home (American Indian or Alaska Native adults: 18.9%; Black adults: 15.2%). Self-protection was a common reason for carrying a firearm (American Indian or Alaska Native adults: 104 [84.9%; 95% CI, 74.1%-91.7%]; Black adults: 350 [88.3%; 95% CI, 82.3%-92.4%]), and a minority of participants cited lack of faith in the police (American Indian or Alaska Native adults: 19 [15.2%; 95% CI, 8.2%-26.7%]; Black adults: 61 [15.4%; 95% CI, 10.3%-21.2%]), indicating potential shifts in public safety dynamics.

**Conclusions and Relevance:**

In this survey study of American Indian or Alaska Native and Black US adults, a substantial percentage of both groups reported living in homes with firearms, storing firearms loaded and unlocked, frequently carrying firearms outside the home, and having been exposed directly and indirectly to gun violence. These findings underscore the need for nuanced public health campaigns and policies and highlight challenges for law enforcement in contexts of racial disparities and changing legal frameworks.

## Key Points

**Question:**

What are the distributions of firearm access, firearm storage, firearm carrying, and gun violence exposure among American Indian or Alaska Native and Black US adults?

**Findings:**

In this nationally representative survey study of 3542 American Indian or Alaska Native and Black US adults, a substantial percentage of both groups reported living in homes with firearms, storing firearms loaded and unlocked, frequently carrying firearms outside the home, and having been exposed directly and indirectly to gun violence.

**Meaning:**

The findings suggest that efforts to address gun violence in both of these communities should recognize and adapt to patterns of firearm access and use.

## Teaser

This survey study examines firearm access, storage, and carrying behaviors and exposure to firearm violence among American Indian or Alaska Native and Black US adults.

## Introduction

In the US, firearms are most commonly owned and used by politically conservative White men residing in suburban and rural areas.^1^ However, the demographic composition of the country is changing, accompanied by an ongoing shift in the landscape of who owns and uses firearms.^2^ Following the onset of the COVID-19 global pandemic in 2020, there was an unprecedented surge in firearm purchasing. An estimated 7.5 million US residents became new firearm owners from January 2019 through April 2021, and most of the purchasers were first-time firearm owners, people from racial and ethnic minority groups, and women.^3^ First-time firearm owners during the purchasing surge were also younger, more politically liberal, and more regionally diverse than those who owned a firearm before 2020.^4^ Evidence suggests that many first-time owners carry their firearms more frequently in public and store at least 1 in an unsecure manner, generating concerns for public safety and child access in the home.^5,6,7^

Despite the insight into new firearm ownership during the COVID-19 purchasing surge, there remains incomplete knowledge of firearm access and behaviors (such as carrying in public and storage) among individuals belonging to racial minority groups. This is particularly true for American Indian or Alaska Native and Black individuals, 2 groups disproportionately impacted by firearm homicide and suicide, respectively.^8^

Beyond ownership, carrying, and storage, research is limited regarding how various segments of American Indian or Alaska Native and Black populations are exposed to firearm violence. This is a notable gap in knowledge given the documented associations of direct, secondary, and community firearm violence exposure with an array of health outcomes likely to influence socioeconomic and racial disparities in well-being.^9,10,11,12,13^ A clearer understanding of within-group variation in firearm behaviors and gun violence exposure related to demographic factors like sex, age, location, and political affiliation in these populations is crucial to inform clinical assessments in support of primary, secondary, and tertiary injury and trauma prevention–targeted interventions for safe firearm use (eg, secure firearm storage campaigns, hospital-based violence intervention programs). To address these gaps in the literature, this study leveraged recent nationally representative data on American Indian or Alaska Native and Black adults to examine demographic differences in firearm access, behavior, and firearm violence exposure in both groups. The study’s goal was not to compare the 2 communities but rather to provide a representative road map for clinicians, scholars, and policy makers to understand how each of these communities is interacting with and being impacted by firearms.

## Methods

### Participants and Procedures

In this survey study, nationally representative samples of adults who identified as American Indian or Alaska Native and/or Black were recruited from KnowledgePanel via Ipsos between April 12 and May 4, 2023. KnowledgePanel is a probability-based web panel developed to be representative of US adults. Individuals could identify with more than 1 racial identity, and as such, some individuals were nested within the results for both American Indian or Alaska Native and Black adults. The Rutgers biomedical and health sciences institutional review board approved the procedures, and all participants provided informed consent prior to completing the protocol. Ipsos compensates survey participants through entry into sweepstakes. The study followed the American Association for Public Opinion Research (AAPOR) reporting guideline.

### Measures

Demographics were assessed using items embedded within KnowledgePanel profile information assessing common demographic information (eg, age, educational level). Political beliefs were assessed by asking participants, “How would you describe your political beliefs?” Answer choices included “highly conservative,” “somewhat conservative,” “moderate,” “somewhat liberal,” and “highly liberal.” Participants self-reported all demographic information.

#### Firearm Access, Storage, and Behavior

Firearm access was assessed by an item that asked, “Is there typically a firearm stored in or around your home?” Types of firearms owned were assessed by an item that asked, “How many of each type of firearm do you currently have in or around your home?” Primary reason for firearm access was assessed via an item that asked, “Which of the following is your primary reason for keeping a firearm at home?” Firearm storage practices were assessed using an item that asked “Please use the following scale to indicate how often you utilize specific firearm storage practices. When answering these questions, consider only times when you are not using your firearm or transporting it to a location for a specific purpose (eg, driving to the firing range). We are interested in how you store your firearms when they are not in use or about to be in use.”

Firearm carrying frequency was assessed by an item that asked, “How frequently do you carry a firearm on your person outside of your home?” Reasons for firearm carrying were assessed with an item that asked, “What are your reasons for carrying your firearm on your person when outside your home? Please select all that apply.” Locations for firearm carrying were assessed with an item that asked, “In which locations do you typically carry your firearm on your person when outside your home? Please select all that apply.”

#### Firearm Violence Exposure

Firearm violence exposure was assessed using a series of 4 items asking whether respondents had ever experienced specific events related to firearm violence. These included the following: “Have you ever been threatened with a firearm by another person?” “Have you ever been shot on purpose by another person with a firearm?” “Do you personally know someone, such as a friend or family member, who has been shot on purpose by another person with a firearm?” and “Have you ever witnessed or heard about someone being shot intentionally by another person with a firearm in your neighborhood?”

### Statistical Analysis

Ipsos developed data weights via 2 steps. Design weights were first computed to reflect selection probabilities. These weights were then weighted to geodemographic distributions (by sex, age, race, census region, metropolitan region, educational level, and household income) obtained from the US Census Bureau’s 2022 Current Population Survey, resulting in weights specific to the American Indian or Alaska Native sample and weights specific to the Black sample. Analyses were weighted based on which group was being analyzed at the time. Weights were trimmed per condition and scaled to sum to the total number of respondents from a particular group. Analyses were conducted using IBM SPSS, version 28.0.1.1 (IBM Corp), and Stata, version 18 (StataCorp LLC).

## Results

Of 7133 surveys fielded, 4232 (59.4%) were completed and 3553 (81.6%) were qualified. Participants were American Indian or Alaskan Native adults (527 [14.9%]; 280 [53.1%] female; 247 [46.9%] male; 396 [75.2%] residing in a metropolitan area) or Black adults (3015 [85.1%]; 1646 [54.6%] female; 1369 [45.4%] male; 2756 [91.4%] residing in a metropolitan area) living in the US. Eighty-nine individuals (2.5% of the total sample) were nested within the results for both American Indian or Alaska Native and Black adults. The Table gives full sample demographic distributions.

**Table.** Sample Demographics for American Indian or Alaska Native and Black Adults

Characteristic	Adults, No. (%)	
	American Indian or Alaska Native (n = 527)	Black (n = 3015)
Age, y		
18-29	84 (15.9)	562 (18.6)
30-44	192 (35.6)	972 (32.2)
45-59	125 (23.7)	728 (24.1)
≥60	126 (23.9)	754 (25.0)
Sex		
Female	280 (53.1)	1646 (54.6)
Male	247 (46.9)	1369 (45.4)
Educational level		
Less than high school	55 (10.5)	177 (5.9)
High school or GED	207 (39.2)	1128 (37.4)
Some college	162 (30.6)	928 (30.8)
Bachelor’s degree or higher	103 (19.6)	782 (25.9)
Annual household income, $		
<10 000	46 (8.6)	372 (12.3)
10 000-24 999	60 (11.4)	249 (8.3)
25 000-49 999	109 (20.7)	640 (21.2)
50 000-74 999	90 (17.1)	523 (17.4)
75 000-99 999	71 (13.5)	373 (12.4)
100,00-149 999	86 (16.3)	458 (15.2)
≥150 000	66 (12.5)	399 (12.2)
Marital status		
Married	256 (48.6)	1079 (35.8)
Widowed	21 (4.0)	149 (5.0)
Divorced	60 (11.4)	364 (12.1)
Separated	12 (2.2)	66 (2.2)
Never married	178 (33.8)	1357 (45.0)
Metropolitan area status		
Nonmetropolitan	131 (24.8)	259 (8.6)
Metropolitan	396 (75.2)	2756 (91.4)
Region of residence		
Northeast	44 (8.3)	514 (17.0)
Midwest	80 (15.2)	484 (16.1)
South	192 (36.4)	1700 (56.4)
West	211 (40.1)	317 (10.5)
Employment status		
Full-time	241 (45.6)	1596 (52.9)
Part-time	69 (13.1)	340 (11.3)
Not working	218 (41.3)	1079 (35.8)
Sexual orientation		
Asexual	3 (0.7)	17 (0.6)
Bisexual	39 (7.4)	175 (5.8)
Gay or lesbian	19 (3.6)	88 (2.9)
Heterosexual	423 (80.3)	2441 (80.9)
Pansexual	9 (1.7)	19 (0.6)
Other	5 (0.9)	52 (1.7)
Did not disclose	27 (5.2)	166 (5.5)
Tribal status		
Enrolled in a tribe	157 (29.8)	1 (<0.1)
Identify with a tribe	288 (54.6)	11 (0.4)

Abbreviation: GED, General Educational Development.

### Black Adults

#### Firearm Access

Nearly one-third of Black adults (909 [30.4%; 95% CI, 28.0%-32.9%]) reported that at least 1 firearm was typically stored in or around their home (eTable 1 in Supplement 1). With respect to sex, 472 Black men (34.7%; 95% CI, 30.6%-39.1%) and 437 Black women (26.8%; 95% CI, 24.2%-29.6%) reported firearm access in the home. Firearm access among Black adults was more common in nonmetropolitan areas (106 adults [41.4%; 95% CI, 33.0%-50.4%]) than in metropolitan areas (803 adults; [29.4%; 95% CI, 26.9%-32.0%]). Geographic variability was notable, with Black adults endorsing firearm access most frequently in the Midwest (173 [36.5%; 95% CI, 30.7%-42.8%]) and South (585 [34.6%; 95% CI, 31.4%-38.0%]) and least frequently in the Northeast (63 [12.3%; 95% CI, 8.6%-17.5%]) (eTable 2 in Supplement 1). Minimal variation was noted by age and political affiliation.

Nearly all Black adult firearms owners (91.1%) endorsed access to handguns. The modal number of handguns owned by Black adults was 1 (451 adults [51.7%; 95% CI, 46.8%-56.6%]), and most Black adults reported keeping no shotguns (581 [66.7%; 95% CI, 61.9%-71.2%]) or rifles (602 [69.1%; 95% CI, 64.4%-73.4%]) in or around their home. Nearly two-thirds of Black adults with firearm access (543 [64.2%; 95% CI, 59.4%-68.8%]) reported that home protection was their primary motive for firearm access.

#### Firearm Storage

Nearly equivalent proportions of Black adults with firearm access endorsed always (304 [39.3%; 95% CI, 34.2%-44.6%]) and never (301 [37.7%; 95% CI, 32.7%-43.0%]) storing at least 1 firearm loaded. This finding varied geographically; in the Northeast, only 16 Black adults (30.7%; 95% CI, 17.2%-48.8%) never stored a firearm loaded compared with 22 (41.0%; 95% CI, 24.1%-60.4%) who always stored a firearm loaded, and in the West, 49 (61.1%; 95% CI, 44.1%-75.8%) never stored a firearm loaded compared with 18 (22.0%; 95% CI, 12.3%-36.2%) who always stored a firearm loaded.

Fairly equivalent proportions of Black individuals reported storing firearms with a locking device (eg, trigger lock, cable lock) never (279 [36.1%; 95% CI, 31.1%-41.5%]) or always (337 [42.4%; 95% CI, 37.3%-47.7%]). Notable exceptions included Black women (never: 103 [28.5%; 95% CI, 23.4%-34.3%]; always: 193 [51.1%; 95% CI, 33.9%-57.3%]), Black adults in the Northeast (never: 12 [22.2%; 95% CI, 11.0%-40.4%]; always: 27 [49.3%; 95% CI, 30.6%-68.1%]), and highly conservative Black individuals (never: 6 [25.3%; 95% CI, 9.6%-51.9%]; always: 15 [56.3%; 95% CI, 32.1%-77.8%]).

Black adults with firearm access more frequently endorsed always storing firearms in a locked location (eg, gun safe, lockbox) (372 [47.9%; 95% CI, 42.6%-53.2%]) than never doing so (223 [28.5%; 95% CI, 23.9%-33.5%]). The only notable exception was among Black adults living in the West (never: 31 [37.6%; 95% CI, 22.2%-56.0%]; always: 32 [39.2%; 95% CI, 25.4%-54.9%]).

A small but meaningful proportion of Black adults (10.0%) reported always or almost always storing at least 1 firearm unlocked in their vehicle. This was particularly common among those aged 18 to 29 years (28.0%), those in the Northeast (19.0%), and those endorsing highly conservative political beliefs (26.5%).

#### Firearm Carrying

A minority of Black adults with firearm access (15.2%) reported always or almost always carrying firearms when outside the home. This was particularly true in the Northeast, where 33.6% reported doing so.

Self-protection was the most frequently endorsed reason among Black adults for carrying firearms (350 [88.3%; 95% CI, 82.3%-92.4%]), with 139 (35.3%; 95% CI, 28.7%-42.6%) endorsing protection of others and 61 (14.9%; 95% CI, 10.3%-21.2%) endorsing lack of faith in police. Protection of others and lack of faith in police were endorsed with greater frequency among some groups, including those aged 18 to 29 years (protection of others: 25 [49.4%; 95% CI, 27.6%-71.4%]; lack of faith in police: 12 [24.4%; 95% CI, 9.8%-49.0%]), those aged 30 to 44 years (protection of others: 58 [56.9%; 95% CI, 42.8%-70.0%]; lack of faith in police: 32 [22.6%; 95% CI, 12.9%-36.5%]), and those in the Northeast (protection of others: 16 [52.3%; 95% CI, 24.6%-78.7%]; lack of faith in police: 9 [28.0%; 95% CI, 7.1%-66.4%]).

Most Black adults (334 [84.4%; 95% CI, 79.5%-88.4%]) endorsed carrying firearms in their vehicles. Nearly half (190 [48.1%; 95% CI, 40.1%-55.5%]) endorsed carrying while walking on the street; 109 (27.6%; 95% CI, 21.5%-34.7%), in retail stores; and 97 (24.6%; 95% CI, 18.9%-31.6%), in restaurants. A notable deviation from this pattern was among Black individuals in the Northeast, where 27 (87.1%; 95% CI, 70.0%-95.9%) endorsed carrying while walking on the street; 23 (75.1%; 95% CI, 50.0%-90.1%), in retail stores; and 23 (75.1%; 95% CI, 50.0%-90.1%), in restaurants.

#### Firearm Violence Exposure

More than 1 in 5 Black adults (650 [21.7%; 95% CI, 19.6%-23.9%]) reported having been threatened with a firearm. A minority reported personally knowing someone who had been shot (1236 [41.3%; 95% CI, 38.8%-43.9%]) and having heard about or witnessed a shooting in their neighborhood (1138 [38.2%; 95% CI, 35.6%-40.7%]). Minimal variability was seen in these variables across demographic groups.

Among Black adults, 80 (2.7%; 95% CI, 2.0%-3.6%) reported having been shot. Variability was noted among several groups of Black adults, including by sex (men: 54 [4.0%; 95% CI, 2.8%-5.7%]; women: 26 [1.6%; 95% CI, 1.0%-2.7%]), metropolitan area status (nonmetropolitan: 15 [6.0%; 95% CI, 3.2%-10.9%]; metropolitan: 65 [2.4%; 95% CI, 1.7%-3.3%]), and geographic region (Northeast: 17 [3.4%; 95% CI, 1.6%-7.0%]; Midwest: 9 [1.9%; 95% CI, 0.9%-4.0%]; South: 51 [3.0%; 95% CI, 2.1%-4.4%]; West: 3 [1.0%; 95% CI, 0.5%-2.2%]).

### American Indian or Alaska Native Adults

#### Firearm Access

Nearly half of American Indian or Alaska Native adults (238 [45.5%; 95% CI, 39.4%-51.7%]) reported that at least 1 firearm was typically stored in or around their home (eTable 3 in Supplement 1). With respect to sex, 133 men (54.2%; 95% CI, 44.6%-63.5%) and 105 women (37.8%; 95% CI, 30.3%-45.9%) reported firearm access in the home. Firearm access increased with age, with 24 of those aged 18 to 29 years (29.1%; 95% CI, 15.2%-48.4%) and 71 of those aged 60 years or older (57.4%; 95% CI, 47.6%-66.7%) reporting access. Over half of those in nonmetropolitan areas (76 [58.7%; 95% CI, 44.7%-71.3%]) and 162 of those in metropolitan areas (41.1%; 95% CI, 34.6%-47.9%) reported firearm access. Geographic variability was notable, with American Indian or Alaska Native adults endorsing firearm access most frequently in the South (113 [59.1%; 95% CI, 49.5%-68.0%]) and least frequently in the Northeast (12 [27.8%; 95% CI, 12.6%-50.7%]) (eTable 4 in Supplement 1). Variability was also notable by political beliefs, with 42 of those who were highly conservative endorsing firearm access (72.6%; 95% CI, 55.2%-85.1%) compared with 15 of those who were highly liberal (28.7%; 95% CI, 16.4%-45.1%).

Most American Indian or Alaska Native adult owners of firearms (86.0%) endorsed access to handguns. The modal number of handguns owned in this group was 2 to 4 (84 adults [40.1%; 95% CI, 31.7%-49.2%]), and most reported keeping at least 1 shotgun (61.0%) or rifle (65.2%) in or around the house. Over half of American Indian or Alaska Native adults with firearm access (115 [57.1%; 95% CI, 48.3%-65.5%]) reported that home protection was their primary motive for firearm access. Notable exceptions included participants in the Midwest, among whom 9 (30.9%; 95% CI, 15.7%-51.7%) reported safety at home as their primary motive, and those with highly liberal beliefs, among whom 3 (21.1%; 95% CI, 7.2%-47.9%) did so.

#### Firearm Storage

Small sample sizes precluded confident interpretations of patterns among storage-related variables across groups of American Indian or Alaska Native adults with firearms. Overall, nearly equivalent percentages of individuals reported always or never storing at least 1 firearm loaded (76 [37.2%; 95% CI, 28.6%-46.5%] vs 85 [43.5%; 95% CI, 34.9%-52.5%]) and with a locking device (77 [40.3%; 95% CI, 31.8%-49.4%] vs 80 [40.0%; 95% CI, 31.6%-49.1%]). It was more common for American Indian or Alaska Native adults to endorse always storing firearms in a locked location (117 [58.4%; 95% CI, 49.5%-66.8%]) than never doing so (48 [23.5%; 95% CI, 17.1%-31.3%]). A small but meaningful proportion (13.0%) endorsed always or almost always storing a firearm unlocked in a vehicle.

#### Firearm Carrying

Small sample sizes precluded confident interpretations of patterns among variables related to firearm carrying across groups of American Indian or Alaska Native adults. A minority of American Indian or Alaska Native adults with firearm access (18.9%) reported always or almost always carrying firearms when outside the home.

Self-protection was the most frequently endorsed reason among American Indian or Alaska Native adults for carrying a firearm (104 [84.9%; 95% CI, 74.1%-91.7%]), with 69 (56.3%; 95% CI, 44.1%-67.9%) endorsing protection of others, 34 (28.0%; 95% CI, 18.6%-39.9%) endorsing hunting or other recreation, and 19 (15.2%; 95% CI, 8.2%-26.7%) endorsing lack of faith in police.

Most of this group (102 [83.2%; 95% CI, 72.9%-90.2%]) endorsed carrying firearms in their vehicles. Nearly half (60 [49.0%; 95% CI, 37.2%-60.9%]) endorsed carrying while walking on the street; 56 (45.4%; 95% CI, 33.8%-57.5%), in retail stores; 49 (39.2%; 95% CI, 28.3%-51.4%), in restaurants; and 46 (37.2%; 95% CI, 26.4%-49.4%), in parks.

#### Firearm Violence Exposure

Nearly one-third of American Indian or Alaska Native adults (158 [30.2%; 95% CI, 24.8%-36.3%]) reported having been threatened with a firearm. There was a notable discrepancy between men (95 [38.6%; 95% CI, 30.1%-48.0%]) and women (63 [22.8%; 95% CI, 16.2%-31.1%]).

Among American Indian or Alaska Native adults, 33 (6.2%; 95% CI, 3.9%-9.8%) reported having been shot. With respect to sex, 22 men (8.8%; 95% CI, 5.2%-14.4%) and 11 women (3.9%; 95% CI, 1.5%-9.9%) reported having been shot. Only 2 living in nonmetropolitan areas (1.2%; 95% CI, 0.4%-3.3%) endorsed having been shot compared with 31 living in metropolitan areas (7.9%; 95% CI, 4.8%-12.6%). Variability was also notable with respect to geographic region (Northeast: 1 [2.7%; 95% CI, 0.4%-17.3%]; Midwest: 3 [3.8%; 95% CI, 1.3%-10.7%]; South: 15 [8.1%; 95% CI, 3.9%-16.0%]; West: 13 [6.2%; 95% CI, 3.0%-12.4%]; 1 individual who had been shot did not report geographic location) and political beliefs (highly conservative: 9 [15.0%; 95% CI, 4.8%-38.5%]; somewhat conservative: 3 [2.4%, 95% CI, 0.8%-6.6%]; moderate: 17 [7.8%; 95% CI, 4.3%-13.7%]; somewhat liberal: 3 [5.0%; 95% CI, 1.3%-18.1%]; highly liberal: 1 [0.4%; 95% CI, 0.0%-2.7%]).

A minority of American Indian or Alaska Native adults endorsed personally knowing someone who had been shot (201 [38.4%; 95% CI, 32.5%-44.5%]) and having heard about or witnessed a shooting in their neighborhood (147 [27.9%; 95% CI, 22.9%-33.5%]). Limited variability was noted across demographic factors.

## Discussion

This study aimed to (1) understand patterns in firearm access and behaviors (ie, ownership, storage, and carrying) among nationally representative samples of American Indian or Alaska Native and Black adults, (2) identify within-group variation in firearm behaviors across geodemographic groups, and (3) quantify lifetime firearm violence exposure within American Indian or Alaska Native and Black populations. Our results produced 4 key findings.

First, a higher proportion of American Indian or Alaska Native adults than Black adults owned a firearm, and firearm ownership in the American Indian or Alaska Native sample increased with age. In line with prior research,^14^ most firearm owners within both samples were male, lived in rural areas, primarily owned a handgun, and cited home protection as the reason for ownership. Second, within both samples, nearly equivalent proportions of firearm owners endorsed always and never storing at least 1 firearm loaded and using a locking device. Most firearm owners in both samples endorsed storing their firearm in a locked location except those who lived in the West, among whom similar proportions endorsed always and never storing their firearm in a locked location. Third, in both samples, most firearm owners rarely or never carried their firearm(s) outside their homes. Among those that endorsed carrying, the main reason was the protection of self and others. Equal proportions of both samples also endorsed lack of faith in police as a reason for carrying. Most respondents who reported carrying outside of the home endorsed carrying firearms in vehicles, and many endorsed carrying while walking on streets.

Fourth, the study found that 21.7% of Black respondents and 30.2% of American Indian or Alaska Native respondents had been threatened with a firearm. A larger proportion of the American Indian or Alaska Native sample than the Black sample had been shot by a firearm in a metropolitan area, while a greater proportion of Black respondents in nonmetropolitan locations indicated being shot than in metropolitan areas. Variability was also seen within the American Indian or Alaska Native sample based on political beliefs. Firearm violence exposure was highest for Black adults in the Northeast and highest for American Indian or Alaska Native adults in the West. In both groups, a notable proportion of individuals endorsed personally knowing someone who had been shot and having heard about or witnessed a shooting in their neighborhood.

Within both samples, there was a notable bifurcation with respect to storing firearms loaded and with locking devices, with nearly equivalent percentages reporting never and always using these storage approaches. This highlights a marked difference between various firearm-owning households with respect to how they view the importance of quick and ready access to firearms and is consistent with the notion that efforts should be made both in clinical interactions and in broad public health efforts to help those with firearm access to understand the dangers associated with unsecured firearms in the home relative to the potential self-protection benefits of quick access.

The more frequent endorsement of gun safes as a storage option also highlights that some of the storage devices most typically dispensed by clinicians and other stakeholders (eg, cable locks) may be less desired by firearm owners and less likely to prompt secure storage behaviors. Recent research has highlighted that firearm owners may prefer a coupon for a discounted gun safe rather than a free cable lock.^15^

Our results contribute to a rapidly expanding body of literature on the nuances of firearm ownership, carrying, and storage among racially diverse populations.^16,17,18^ This evidence is crucial to inform tailored messaging related to firearm safety and secure storage programs (eg, Be SMART, Means Matter, Lock It Up, and Gun Shop Project). Our results showed that most American Indian or Alaska Native and Black respondents stored their personally owned firearms in a locked location, but mixed practices on storing firearms loaded and/or using a locking device reveal ample avenues for continued education and programming on firearm locking devices. For instance, recent research suggests that many firearm owners do not prefer to use cable locks and might instead prefer biometric gun safes or in-vehicle lockboxes.^15^ Our findings on the extent of different types of firearm violence exposure underscore the need for trauma-informed modules embedded within firearm safety and secure storage programs.

### Limitations

There are several limitations to note for this study. First, we relied on self-reported data, and recent research suggests that certain groups may underreport their number of firearms, firearm carrying and storage behaviors, and/or firearm violence exposures.^19^ Second, our data are cross-sectional, and our analyses were unable to account for any changes in firearm ownership or behaviors over time. Third, we could not confidently interpret patterns across groups related to firearm storage methods due to small sample sizes in individual cells. Fourth, although KnowledgePanel recruitment does not require that individuals be housed and unincarcerated, undoubtedly few if any participants were unhoused or incarcerated, which could have implications for the generalizability of our findings. Fifth, the time sequence between violence exposure and firearm access and use behaviors is unknown, precluding any examination of the potential association of violence exposure with firearm acquisition and use. Finally, our results are only representative of American Indian or Alaska Native and Black populations in the US. Future studies should seek to replicate these results among other racial and ethnic groups in the US, including Asian, Hispanic, and White individuals.

## Conclusions

This survey study provided insight into geodemographic differences in firearm behaviors and violence exposure among American Indian or Alaska Native and Black adults in the US. The results revealed roughly equivalent firearm storage patterns in the American Indian or Alaska Native and Black populations, protection as a main reason for owning or carrying a firearm, and common experiences of both indirect and direct firearm violence exposure. The descriptive information presented here has implications for informing public health campaigns and policies to promote safe firearm use and prevent inequitable exposure to firearm violence.

## Notes

Supplement 1. **eTable 1.** Sex, Age, and Metropolitan Area Differences in Firearm Access, Behaviors, and Exposure Among Black Adults **eTable 2.** Regional and Political Group Differences in Firearm Access, Behaviors, and Exposure Among Black Adults **eTable 3.** Sex, Age, and Metropolitan Area Differences in Firearm Access, Behaviors, and Exposure Among American Indian or Alaska Native Adults **eTable 4.** Regional and Political Group Differences in Firearm Access, Behaviors, and Exposure Among American Indian or Alaska Native Adults

Supplement 2.
Data Sharing Statement
