# Supplementary material for: Firearm Access and Gun Violence Exposure Among American Indian or Alaska Native and Black Adults
Source: JAMA Netw Open. 2024 Mar 4;7(3):e240073. doi: 10.1001/jamanetworkopen.2024.0073 (PMC10912965; doi:10.1001/jamanetworkopen.2024.0073)
Supplement: Supplement 1. — eTable 1. Sex, Age, and Metropolitan Area Differences in Firearm Access, Behaviors, and Exposure Among Black Adults eTable 2. Regional and Political Group Differences in Firearm Access, Behaviors, and Exposure Among Black Adults eTable 3. Sex, Age, and Metropolitan Area Differences in Firearm Access, Behaviors, and Exposure Among American Indian or Alaska Native Adults eTable 4. Regional and Political Group Differences in Firearm Access, Behaviors, and Exposure Among American Indian or Alaska Native Adults [file jamanetwopen-e240073-s001.pdf]

# Supplemental Online Content

Anestis MD, Moceri-Brooks J, Ziminski D, Barnes RT, Semenza D. Firearm access and gun violence exposure among American Indian or Alaska Native and Black adults. *JAMA Netw Open*. 2024;7(3):e240073. doi:10.1001/jamanetworkopen.2024.0073

**eTable 1.** Sex, Age, and Metropolitan Area Differences in Firearm Access, Behaviors, and Exposure Among Black Adults

**eTable 2.** Regional and Political Group Differences in Firearm Access, Behaviors, and Exposure Among Black Adults

**eTable 3.** Sex, Age, and Metropolitan Area Differences in Firearm Access, Behaviors, and Exposure Among American Indian or Alaska Native Adults

**eTable 4.** Regional and Political Group Differences in Firearm Access, Behaviors, and Exposure Among American Indian or Alaska Native Adults

This supplemental material has been provided by the authors to give readers additional information about their work.

© 2024 Anestis MD et al. *JAMA Network Open*.

**eTable 1.** Sex, Age, and Metropolitan Area Differences in Firearm Access, Behaviors, and Exposure Among Black Adults

| Characteristic | Black adults, No. (%) [95% CI] | | | | | | | | |
|---|---|---|---|---|---|---|---|---|---|
| | Total | Sex | | Age, y | | | | Metropolitan area status | |
| | | Female | Male | 18-29 | 30-44 | 45-59 | ≥60 | Nonmetropolitan | Metropolitan |
| **Firearm in or around home** | | | | | | | | | |
| Any | 909 (30.4) [28.0-32.9] | 437 (26.8) [24.2-29.6] | 472 (34.7) [30.6-39.1] | 133 (23.9) [17.6-31.6] | 278 (28.8) [24.4-33.6] | 232 (32.0) [28.1-36.3] | 267 (35.8) [32.0-39.7] | 106 (41.4) [33.0-50.4] | 803 (29.4) [26.9-32.0] |
| Handguns, No. | | | | | | | | | |
| 0 | 78 (9.0) [6.4-12.2] | 39 (9.2) [6.4-13.0] | 39 (8.7) [5.1-14.5] | 14 (10.4) [3.8-25.3] | 36 (13.8) [8.2-22.2] | 10 (4.6) [2.4-8.6] | 18 (7.0) [4.6-10.5] | 20 (19.0) [11.5-29.7] | 58 (7.6) [5.1-11.2] |
| 1 | 451 (51.7) [46.8-56.6] | 232 (55.2) [49.3-60.9] | 219 (48.5) [40.8-56.2] | 73 (56.7) [39.7-72.2] | 151 (57.2) [47.5-66.4] | 107 (48.0) [40.2-55.8] | 120 (46.7) [39.6-53.9] | 36 (34.5) [22.9-48.3] | 414 (54.0) [48.8-59.2] |
| 2-4 | 312 (35.8) [31.4-40.5] | 144 (34.2) [20.0-39.9] | 168 (37.3) [30.3-44.8] | 39 (29.8) [17.3-46.3] | 72 (27.4) [20.0-36.1] | 89 (39.9) [32.4-48.0] | 113 (43.9) [36.8-51.4] | 43 (40.9) [28.1-55.0] | 269 (35.1) [30.4-40.1] |
| ≥5 | 31 (3.6) [2.1-5.8] | 6 (1.4) [0.5-3.7] | 25 (5.6) [3.1-9.7] | 4 (3.1) [0.4-18.9] | 4 (1.6) [0.4-6.8] | 17 (7.5) [3.9-14.0] | 6 (2.4) [1.2-4.5] | 6 (5.6) [2.2-13.5] | 25 (3.3) [1.8-5.8] |
| Shotguns, No. | | | | | | | | | |
| 0 | 581 (66.7) [61.9-71.2] | 307 (73.2) [67.5-78.3] | 274 (60.7) [53.0-67.9] | 94 (72.7) [55.3-85.2] | 184 (70.0) [60.2-78.3] | 147 (66.0) [57.6-73.4] | 156 (61.0) [53.6-67.9] | 53 (50.9) [37.6-64.2] | 528 (68.9) [63.8-73.5] |
| 1 | 195 (22.4) [18.7-26.6] | 82 (19.5) [15.4-24.5] | 113 (25.1) [19.2-32.0] | 16 (12.2) [5.4-25.4] | 65 (24.8) [17.1-34.6] | 55 (24.8) [18.2-32.7] | 59 (23.0) [17.9-29.1] | 31 (29.2) [18.3-43.2] | 165 (21.5) [17.6-25.9] |
| 2-4 | 86 (9.8) [7.1-13.5] | 29 (6.9) [3.9-11.7] | 57 (12.5) [8.3-18.5] | 16 (12.0) [4.1-30.5] | 14 (5.2) [2.4-10.6] | 20 (9.0) [4.9-15.9] | 36 (14.2) [9.1-21.4] | 17 (16.1) [7.3-31.9] | 69 (8.9) [6.2-12.6] |
| ≥5 | 10 (1.1) [0.3-3.5] | 2 (0.4) [0.1-1.5] | 8 (1.8) [0.4-6.7] | 4 (3.1) [0.4-18.9] | 0 | 1 (0.3) [0.0-2.2] | 5 (1.9) [0.4-9.0] | 4 (3.7) [0.5-21.9] | 6 (0.7) [0.2-3.1] |
| Rifles, No. | | | | | | | | | |
| 0 | 602 (69.1) [64.4-73.4] | 310 (73.7) [68.3-78.5] | 292 (64.7) [57.1-71.5] | 95 (73.4) [55.8-85.8] | 193 (73.4) [64.3-80.9] | 143 (64.3) [55.9-71.8] | 170 (66.5) [59.4-73.0] | 61 (58.8) [45.5-70.9] | 540 (70.5) [65.4-75.0] |
| 1 | 187 (21.4) [17.8-25.6] | 88 (20.9) [16.5-26.2] | 99 (21.9) [16.4-28.6] | 22 (17.2) [7.8-33.7] | 51 (19.5) [13.1-28.0] | 53 (24.0) [17.4-32.2] | 60 (23.3) [17.8-29.9] | 30 (28.8) [19.1-41.1] | 157 (20.4) [16.5-25.0] |
| 2-4 | 72 (8.3) [5.9-11.4] | 19 (4.5) [2.9-6.9] | 53 (11.8) [7.7-17.6] | 8 (6.4) [1.5-23.2] | 16 (5.9) [2.7-12.5] | 23 (10.5) [6.2-17.3] | 25 (9.7) [6.1-15.2] | 10 (9.7) [3.9-22.1] | 62 (8.1) [5.7-11.4] |
| ≥5 | 11 (1.2) [1.0-2.9] | 4 (0.8) [0.2-2.7] | 7 (1.6) [0.5-5.0] | 4 (3.1) [0.4-18.9] | 3 (1.2) [0.3-4.2] | 3 (1.2) [0.4-3.7] | 1 (0.4) [0.1-1.7] | 3 (2.7) [0.7-10.3] | 8 (1.0) [0.4-3.0] |
| **Primary firearm reason** | | | | | | | | | |
| Gift or inheritance | 64 (7.5) [5.3-10.5] | 29 (7.0) [5.1-9.6] | 35 (7.9) [4.5-13.7] | 10 (8.1) [2.0-27.5] | 13 (4.9) [1.8-12.9] | 14 (6.4) [3.8-10.5] | 27 (10.8) [7.6-15.1] | 8 (7.8) [4.0-14.6] | 56 (7.5) [5.1-10.8] |

© 2024 Anestis MD et al. *JAMA Network Open.*

| Characteristic | Black adults, No. (%) [95% CI] | | | | | | | | |
|---|---|---|---|---|---|---|---|---|---|
| | Total | Sex | | Age, y | | | | Metropolitan area status | |
| | | Female | Male | 18-29 | 30-44 | 45-59 | ≥60 | Nonmetropolitan | Metropolitan |
| Safety at home | 543 (64.2) [59.4-68.8] | 227 (55.9) [49.9-61.7] | 316 (71.9) [64.4-78.4] | 66 (54.4) [36.5-71.3] | 169 (65.7) [56.1-74.2] | 156 (72.9) [65.9-78.9] | 152 (60.1) [53.0-66.8] | 63 (62.3) [49.0-74.0] | 481 (64.5) [59.2-69.4] |
| Safety away from home | 53 (6.3) [4.3-9.1] | 23 (5.8) [3.6-9.0] | 30 (6.8) [3.8-11.8] | 2 (1.4) [0.3-5.6] | 26 (10.2) [5.5-18.3] | 8 (3.9) [2.0-7.5] | 17 (6.7) [3.7-11.9] | 6 (6.2) [1.9-18.0] | 47 (6.3) [4.2-9.4] |
| Hunting | 17 (2.1) [1.1-3.9] | 5 (1.4) [0.5-3.3] | 12 (2.7) [1.1-6.3] | 0 | 7 (2.9) [1.1-7.5] | 1 (0.1) [0.0-0.1] | 10 (3.9) [1.6-9.1] | 12 (12.4) [5.5-25.7] | 5 (0.7) [0.3-1.7] |
| Other recreation | 23 (2.8) [1.6-4.6] | 5 (1.3) [0.4-4.0] | 18 (4.1) [2.3-7.3] | 7 (6.1) [1.9-17.8] | 4 (1.7) [0.4-6.7] | 5 (2.5) [1.0-6.2] | 6 (2.4) [1.2-4.7] | 1 (1.4) [0.3-5.5] | 22 (2.9) [1.7-5.1] |
| Belongs to someone else | 114 (13.4) [10.5-17.1] | 93 (22.8) [18.2-28.2] | 21 (4.8) [18.2-28.2] | 27 (22.5) [10.6-41.7] | 28 (10.8) [6.6-17.1] | 23 (10.6) [7.1-15.6] | 36 (14.2) [10.3-19.4] | 8 (8.3) [4.5-15.0] | 105 (14.1) [10.8-18.2] |
| **Firearm storage**[a] | | | | | | | | | |
| ≥1 Loaded | | | | | | | | | |
| Never | 301 (37.7) [32.7-43.0] | 161 (41.8) [35.8-48.2] | 40 (34.3) [26.9-42.4] | 41 (39.4) [22.5-59.2] | 94 (40.0) [28.6-51.3] | 74 (35.0) [27.8-43.0] | 92 (37.2) [30.5-44.6] | 38 (37.2) [24.3-52.3] | 263 (37.8) [32.5-43.4] |
| Rarely | 66 (8.5) [5.9-12.1] | 35 (9.9) [6.7-14.3] | 31 (7.3) [3.9-13.5] | 6 (6.1) [1.6-21.0] | 24 (11.3) [5.6-21.4] | 14 (6.0) [3.4-10.3] | 22 (9.4) [5.4-15.7] | 11 (11.9) [6.3-21.5] | 55 (8.0) [5.3-12.0] |
| Occasionally | 34 (4.7) [3.1-6.9] | 18 (5.3) [3.3-8.5] | 16 (4.1) [2.1-7.8] | 2 (2.0) [0.4-8.4] | 8 (4.0) [1.7-9.4] | 9 (4.4) [2.2-8.6] | 15 (6.8) [3.5-12.4] | 6 (6.1) [2.1-16.2] | 29 (4.5) [2.9-6.8] |
| Often | 27 (3.4) [1.8-6.3] | 9 (2.0) [1.1-3.6] | 18 (4.4) [1.9-10.1] | 10 (9.7) [2.4-32.0] | 5 (1.5) [0.6-4.2] | 6 (3.1) [1.4-6.6] | 6 (2.3) [1.1-4.8] | 1 (0.3) [0.0-1.9] | 26 (3.8) [2.0-7.1] |
| Almost always | 50 (6.5) [4.5-9.4] | 24 (6.7) [4.2-10.6] | 26 (6.4) [3.6-11.1] | 6 (5.4) [1.6-16.8] | 17 (7.4) [3.7-14.3] | 3 (6.2) [3.1-12.2] | 15 (6.5) [3.4-12.1] | 9 (9.9) [3.7-24.1] | 41 (6.1) [4.1-9.0] |
| Always | 304 (39.3) [34.2-44.6] | 120 (34.2) [28.5-40.4] | 184 (43.5) [35.6-51.8] | 39 (37.6) [21.7-56.7] | 83 (35.8) [25.7-47.3] | 96 (45.3) [37.1-53.9] | 86 (37.8) [30.4-45.8] | 33 (35.0) [21.8-50.1] | 271 (39.9) [34.5-45.7] |
| ≥1 With locking device | | | | | | | | | |
| Never | 279 (36.1) [31.1-41.5] | 103 (28.5) [23.4-34.3] | 176 (42.4) [34.5-50.7] | 39 (35.2) [19.5-54.9] | 78 (35.8) [25.5-47.5] | 71 (33.8] (25.9-42.8] | 91 (38.9) [31.8-46.6] | 31 (33.9) [21.6-48.9] | 248 (36.4) [31.0-42.2] |
| Rarely | 53 (6.2) [4.2-9.2] | 19 (4.9) [3.0-8.0] | 34 (7.4) [4.3-12.6] | 12 (9.5) [2.6-29.4] | 11 (4.2) [1.6-10.6] | 15 (6.9) [4.2-11.2] | 14 (6.2) [3.6-10.5] | 7 (8.1) [3.4-17.8] | 45 (6.0) [3.9-9.3] |
| Occasionally | 32 (4.1) [2.6-6.3] | 19 (5.4) [3.1-9.3] | 13 (3.0) [1.5-6.0] | 2 (2.4) [0.3-15.2] | 4 (1.7) [0.6-5.0] | 15 (6.4) [3.4-11.0] | 11 (5.0) [2.4-10.0] | 4 (4.4) [1.4-13.0] | 27 (4.0) [2.5-6.5] |
| Often | 26 (3.4) [5.1-11.4] | 14 (4.0) [2.3-7.1] | 12 (2.9) [1.4-6.0] | 0 | 6 (3.0) [1.2-7.1] | 11 (5.2) [2.4-10.8] | 8 (3.9) [1.9-7.8] | 4 (4.5) [1.7-11.5] | 21 (3.3) [2.0-5.4] |
| Almost always | 59 (7.7) [5.1-11.4] | 21 (6.0) [3.7-9.7] | 38 (9.1] (5.1-15.6] | 7 (6.8) [2.0-20.4] | 17 (8.2) [3.1-20.1] | 14 (6.4) [3.5-11.2] | 20 (8.9) [5.1-15.3] | 17 (18.1) [7.8-36.7] | 42 (6.2) [4.0-10.0] |

© 2024 Anestis MD et al. *JAMA Network Open.*

| Characteristic | Black adults, No. (%) [95% CI] | | | | | | | | |
|---|---|---|---|---|---|---|---|---|---|
| | Total | Sex | | Age, y | | | | Metropolitan area status | |
| | | Female | Male | 18-29 | 30-44 | 45-59 | ≥60 | Nonmetropolitan | Metropolitan |
| Always | 337 (42.4) [37.3-47.7] | 193 (51.1) [33.9-57.3] | 144 (35.2) [27.9-43.3] | 48 (46.1) [28.4-64.9] | 103 (47.2) [36.4-58.3] | 90 (41.3) [33.6-49.5] | 96 (37.1) [29.9-45.0] | 30 (31.0) [17.7-45.0] | 307 (44.1) [38.5-49.7] |
| ≥1 In locked location | | | | | | | | | |
| Never | 223 (28.5) [23.9-33.5] | 92 (24.6) [19.8-30.1] | 131 (31.7) [24.6-39.8] | 29 (25.5) [12.5-45.2] | 51 (22.5) [14.5-33.3] | 58 (27.6) [20.1-35.6] | 85 (36.5) [29.4-44.2] | 30 (30.7) [18.5-46.4] | 193 (28.2) [23.4-33.5] |
| Rarely | 47 (6.0) [4.0-9.0] | 22 (5.9) [3.8-9.2] | 25 (6.1) [3.3-11.3] | 15 (12.7) [4.4-31.3] | 7 (3.1) [1.3-7.3] | 11 (5.6) [3.0-10.0] | 14 (6.1) [3.6-10.3] | 5 (5.6) [2.7-11.2] | 42 (6.1) [3.9-9.4] |
| Occasionally | 32 (4.2) [2.6-6.7] | 13 (3.6) [1.8-7.1] | 19 (4.7) [2.5-8.8] | 1 (0.6) [0.1-4.3] | 7 (3.1) [1.4-6.9] | 11 (5.2) [2.7-9.8] | 14 (6.0) [2.5-13.9] | 5 (5.6) [2.7-11.2] | 27 (6.1) [3.9-9.4] |
| Often | 32 (3.9) [2.4-6.2] | 12 (3.6) [1.8-6.9] | 20 (4.2) [2.2-7.9] | 3 (2.8) [0.4-17.6] | 7 (2.1) [0.8-5.5] | 13 (5.9) [2.8-12.1] | 9 (4.3) [2.0-9.0] | 9 (4.8) [1.2-17.0] | 24 (4.1) [2.5-6.8] |
| Almost always | 72 (9.5) [6.4-13.7] | 30 (8.6) [5.4-13.4] | 42 (10.2) [5.7-17.6] | 16 (15.3) [5.8-34.8] | 23 (10.5) [4.5-22.6] | 17 (7.4) [4.1-13.0] | 17 (7.6) [4.3-13.2] | 5 (5.6) [1.6-17.8] | 67 (10.0) [6.6-14.8] |
| Always | 372 (47.9) [42.6-53.2] | 196 (53.7) [47.4-59.9] | 176 (43.1) [35.2-51.3] | 45 (43.1) [25.8-62.2] | 131 (58.7) [47.3-69.2] | 106 (48.4) [40.2-56.7] | 89 (39.5) [32.4-47.1] | 41 (43.8) [30.1-58.6] | 330 (48.5) [42.8-54.2] |
| ≥1 In vehicle unlocked | | | | | | | | | |
| Never | 594 (75.0) [69.7-78.9] | 279 (72.0) [65.4-77.8] | 315 (76.7) [69.5-82.6] | 62 (55.5) [36.8-72.8] | 159 (70.4) [60.1-79.0] | 176 (82.8) [76.6-87.7] | 196 (80.0) [72.7-85.7] | 67 (72.3) [58.6-82.8] | 526 (74.9) [69.6-79.5] |
| Rarely | 63 (8.3) [5.9-11.6] | 37 (10.9) [6.9-16.8] | 26 (6.1) [3.6-10.1] | 11 (10.5) [3.0-30.8] | 17 (7.1) [3.8-13.0] | 14 (6.8) [3.8-11.7] | 22 (9.9) [6.0-15.9] | 5 (5.6) [2.3-13.1] | 58 (8.7) [6.0-12.4] |
| Occasionally | 36 (4.7) [3.0-7.4] | 11 (3.4) [1.7-6.6] | 25 (5.9) [3.2-10.5] | 4 (4.0) [0.9-16.4] | 13 (6.3) [2.5-15.0] | 5 (1.4) [0.6-3.4] | 14 (2.8) [0.7-10.4] | 8 (8.8) [4.0-18.6] | 29 (4.1) [2.4-7.1] |
| Often | 20 (2.4) [1.3-4.5] | 8 (1.6) [0.1-3.8] | 12 (3.0) [1.3-6.9] | 2 (1.9) [0.4-8.4] | 9 (3.2) [1.2-8.1] | 3 (1.4) [0.36 3.4] | 6 (2.8) [0.7,10.4] | 5 (5.0) [0.9-22.8] | 16 (2.0) [1.1-3.8] |
| Almost always | 18 (2.5) [1.3-4.9] | 9 (2.7) [1.3-5.6] | 9 (2.3) [0.7-7.0] | 2 (8.7) [2.7-24.8] | 9 (3.0) [1.1-7.7] | 3 (1.1) [0.3-3.5] | 6 (5.0) [0.2-1.5] | 2 (2.5) [0.6-10.1] | 16 (5.9) [2.4-13.8] |
| Always | 59 (7.5) [5.0-10.9] | 33 (9.3) [5.9-14.5] | 26 (5.9) [3.0-11.3] | 20 (19.3) [8.6-37.6] | 21 (10.0) [8.6-37.6] | 15 (5.4) [3.0-9.5] | 3 (1.4) [0.6-3.3] | 5 (5.9) [2.4-13.8] | 54 (7.7) [5.0-11.6] |
| **Firearm carrying** | | | | | | | | | |
| Frequency | | | | | | | | | |
| Never | 454 (53.3) [48.3-58.2] | 252 (62.1) [56.2-67.7] | 202 (45.2) [37.7-53.1] | 72 (59.4) [42.1-74.6] | 121 (46.9) [37.2-56.9] | 107 (48.5) [40.6-56.4] | 154 (61.1) [53.5-68.1] | 52 (52.4) [38.6-65.9] | 401 (53.4) [48.1-58.7] |
| Rarely | 134 (15.7) [12.6-19.6] | 57 (14.1) [10.9-18.2] | 77 (17.2) [12.2-23.8] | 11 (9.4) [4.1-20.1] | 48 (18.7) [12.0-28.0] | 41 (18.7) [12.0-28.0] | 33 (13.2) [9.6-18.0] | 11 (10.8) [6.1-18.4] | 123 (16.4) [12.9-20.7] |

© 2024 Anestis MD et al. *JAMA Network Open.*

| Characteristic | Black adults, No. (%) [95% CI] | | | | | | | | |
|---|---|---|---|---|---|---|---|---|---|
| | Total | Sex | | Age, y | | | | Metropolitan area status | |
| | | Female | Male | 18-29 | 30-44 | 45-59 | ≥60 | Nonmetropolitan | Metropolitan |
| Sometimes | 59 (11.1) [8.6-14.3] | 95 (8.8) [6.1-12.5] | 36 (13.2) [9.2-18.7] | 13 (11.0) [4.3-25.0] | 21 (8.0) [4.7-13.4] | 30 (13.5) [9.4-19.2] | 31 (12.2) [7.5-19.3] | 22 (21.5) [11.5-36.8] | 73 (9.7) [7.4-12.8] |
| Frequently | 40 (4.7) [3.1-7.0] | 10 (2.4) [1.3-4.5] | 30 (6.8) [4.1-11.1] | 1 (0.5) [0.1-3.8] | 15 (5.7) [2.9-11.0] | 17 (77.4) [4.2-13.8] | 8 (3.0) [1.1-7.9] | 4 (3.7) [0.9-15.0] | 36 (4.8) [3.2-7.3] |
| Almost always | 60 (7.1) [4.6-10.6] | 16 (4.0) [2.4-6.8] | 44 (9.8) [5.7-16.4] | 10 (8.3) [2.4-25.4] | 23 (9.0) [4.0-18.9] | 17 (7.6) [4.1-13.4] | 10 (4.0) [2.1-7.7] | 3 (2.6) [0.8-8.2] | 57 (7.6) [4.9-11.7] |
| Always | 69 (8.1) [5.6-11.5] | 35 (8.6) [5.3-13.6] | 34 (7.6) [4.4-13.1] | 14 (11.5) [4.9-24.6] | 30 (11.6) [6.5-19.9] | 9 (4.0) [1.4-10.8] | 16 (6.4) [3.1-12.7] | 9 (8.9) [2.9-24.1] | 60 (8.0) [5.4-11.6] |
| Reason | | | | | | | | | |
| Lack of faith in police | 61 (14.9) [10.3-21.2] | 16 (9.1) [4.2-18.8] | 45 (18.6) [12.1-27.5] | 12 (24.4) [9.8-49.0] | 32 (22.6) [12.9-36.5] | 8 (7.1) [3.1-15.3] | 9 (8.9) [4.1-18.1] | 5 (11.5) [4.4-26.8] | 56 (15.4) [10.4-22.4] |
| Required for job | 29 (7.5) [4.5-12.3] | 9 (6.2) [2.8-13.3] | 20 (8.3) [4.3-15.3] | 4 (8.1) [1.1-40.0] | 15 (11.1) [5.3-21.7] | 9 (8.4) [3.9-17.0] | 1 (1.4) [0.4-4.5] | 3 (7.3) [2.1-22.1] | 26 (7.5) [4.3-12.8] |
| Expression of freedom | 37 (9.2) [5.5-14.8] | 6 (3.7) [1.5-8.9] | 31 (12.5) [7.1-21.2] | 8 (17.2) [6.2-39.3] | 15 (11.3) [4.7-25.3] | 10 (11.4) [4.7-25.3] | 2 (2.2) [0.7-6.4] | 1 (2.2) [0.5-9.3] | 35 (10.1) [6.0-16.4] |
| Self-protection | 350 (88.3) [82.3-92.4] | 135 (88.4) [80.4-93.4] | 215 (88.2) [70.3-93.6] | 43 (87.0) [59.5-96.8] | 117 (85.7) [71.7-93.4] | 101 (89.3) [79.3-94.7] | 90 (91.3) [83.1-95.7] | 40 (84.5) [64.3-94.3] | 311 (88.8) [92.3-93.1] |
| Protect others | 139 (35.3) [28.7-42.6] | 40 (26.8) [19.3-36.0] | 99 (40.6) [31.1-50.8] | 25 (49.4) [27.6-71.4] | 58 (56.9) [42.8-70.0] | 36 (32.0) [22.1-43.9] | 20 (20.8 (13.3-31.1] | 13 (27.0) [13.1-47.6] | 127 (36.4) [29.3-44.2] |
| Hunting or recreation | 31 (7.9) [4.9-12.3] | 6 (3.7) [1.5-8.5] | 10.3 (25) [6.1-17.3] | 7 (14.4) [4.3-38.4] | 4 (3.2) [0.9-10.4] | 10 (9.0) [4.1-18.8] | 9 (9.6) [4.6-18.7] | 2 (5.3) [1.6-15.7] | 29 (8.2) [5.0-13.2] |
| Location | | | | | | | | | |
| Vehicle | 334 (84.4) [79.5-88.4] | 123 (81.1) [73.1-87.1] | 211 (81.0) [72.9-87.1] | 43 (87.0) [69.0-95.2] | 112 (83.1) [72.4-90.3] | 93 (82.1) [72.2-90.0] | 86 (87.7) [80.2-92.6] | 40 (84.3) [69.5-92.7] | 294 (84.5) [79.0-88.7] |
| Walking on the street | 190 (48.1) [40.1-55.5] | 56 (37.0) [28.4-46.5] | 134 (55.0) [44.8-64.8] | 25 (51.7) [29.7-73.2] | 70 (51.7) [38.0-65.2] | 52 (45.6) [34.1-57.5] | 43 (44.3) [32.0-57.4] | 15 (32.1) [15.0-55.8] | 175 (50.3) [42.6-58.0] |
| Retail stores | 109 (27.6) [21.5-34.7] | 29 (19.1) [12.9-27.2] | 80 (32.9) [24.0-43.2] | 12 (24.7) [10.2-48.7] | 49 (36.5) [24.2-50.9] | 27 (24.2) [15.7-35.3] | 20 (20.8) [12.2-33.2] | 5 (9.7) [4.0-21.5] | 104 (30.1) [23.3-37.8] |
| Restaurants | 97 (24.7) [18.9-31.6] | 27 (18.0) [12.1-26.0] | 70 (28.9) [20.6-39.0] | 9 (17.9) [8.1-38.4] | 47 (34.5) [22.4-49.1] | 27 (23.7) [15.4-34.6] | 16 (15.9) [8.7-27.3] | 5 (10.6) [4.6-22.5] | 93 (26.7) [20.3-34.3] |
| Places of worship | 43 (10.9) [7.5-15.5] | 16 (10.4) [6.0-17.4] | 27 (11.1) [6.7-17.9] | 6 (13.0) [4.5-32.2] | 11 (7.8) [3.6-16.1] | 11 (10.0) [4.7-20.0] | 15 (15.0) [8.2-25.8] | 6 (12.0) [4.2-30.0] | 37 (10.7) [7.1-15.7] |
| Parks | 104 (26.1) [20.1-33.2] | 26 (16.9) [11.1-24.9] | 78 (31.9) [23.1-42.1] | 14 (29.1) [12.9-53.4] | 47 (34.8) [22.7-49.3] | 27 (23.7) [15.6-34.4] | 15 (15.4) [8.4-26.6] | 5 (10.0) [4.2-22.0] | 99 (28.3) [21.7-36.1] |
| Others' homes | 67 (16.9) [12.0-23.5] | 16 (10.4) [6.0-17.3] | 51 (21.0) [13.7-30.8] | 7 (13.6) [4.8-33.0] | 35 (25.9) [15.0-41.0] | 16 (14.0) [8.0-23.2] | 10 (9.7) [4.7-18.9] | 5 (10.6) [4.0-25.4] | 62 (17.8) [12.3-25.0] |

© 2024 Anestis MD et al. *JAMA Network Open.*

| Characteristic | Black adults, No. (%) [95% CI] | | | | | | | | |
|---|---|---|---|---|---|---|---|---|---|
| | Total | Sex | | Age, y | | | | Metropolitan area status | |
| | | Female | Male | 18-29 | 30-44 | 45-59 | ≥60 | Nonmetropolitan | Metropolitan |
| **Firearm violence exposure** | | | | | | | | | |
| Threatened with firearm | 650 (21.7) [19.6-23.9] | 246 (15.1) [13.0-17.3] | 404 (29.6) [25.9-33.6] | 95 (17.2) [11.9-24.1] | 218 (22.5) [18.8-26.8] | 171 (23.5) [19.9-27.6] | 166 (22.1) [19.0-25.7] | 60 (23.5) [16.8-31.7] | 590 (21.5) [19.4-23.8] |
| Shot | 80 (2.7) [2.0-3.6] | 26 (1.6) [1.0-2.7] | 54 (4.0) [2.8-5.7] | 14 (2.6) [1.1-5.9] | 29 (3.1) [1.8-5.3] | 24 (3.3) [2.1-5.2] | 13 (1.7) [1.1-2.7] | 15 (6.0) [3.2-10.9] | 65 (2.4) [1.7-3.3] |
| Friend or family member shot | 1236 (41.3) [38.8-43.9] | 646 (40.0) [36.7-42.7] | 590 (43.3) [39.0-47.7] | 191 (34.6) [27.5-42.4] | 398 (41.4) [36.7-46.4] | 334 (46.0) [41.6-50.5] | 313 (41.7) [37.9-45.7] | 112 (44.2) [35.4-54.4] | 1124 (41.1) [38.4-43.8] |
| Shooting in neighborhood | 1138 (38.2) [35.6-40.7] | 593 (36.5) [33.6-39.5] | 545 (40.1) [35.9-44.5] | 206 (37.5) [30.1-45.4] | 378 (39.5) [34.7-44.4] | 272 (37.5) [33.4-41.8] | 282 (37.7) [33.9-41.6] | 108 (42.4) [33.7-51.6] | 1029 (37.8) [35.2-40.4] |

[a]Rarely, 1% to 25% of the time; occasionally, 26% to 50%; often, 51% to 75%; almost always, 76% to 99%.

© 2024 Anestis MD et al. *JAMA Network Open.*

**eTable 2.** Regional and Political Group Differences in Firearm Access, Behaviors, and Exposure Among Black Adults

| Characteristic | Black adults, No. (%) [95% CI] | | | | | | | | |
|---|---|---|---|---|---|---|---|---|---|
| | Region | | | | Political beliefs | | | | |
| | Northeast | Midwest | South | West | Highly conservative | Somewhat conservative | Moderate | Somewhat liberal | Highly liberal |
| **Firearm in or around home** | | | | | | | | | |
| Any | 63 (12.3) [8.6-17.5] | 173 (36.5) [30.7-42.8] | 585 (34.6) [31.4-38.0] | 88 (27.8) [21.1-35.8] | 34 (28.0) [18.9-39.4] | 124 (36.9) [29.8-44.7] | 503 (31.1) [27.8-34.6] | 171 (29.6) [24.6-35.1] | 57 (20.1) [14.7-26.9] |
| Handguns, No. | | | | | | | | | |
| 0 | 8 (12.2) [3.1-37.3] | 15 (8.8) [4.8-15.7] | 51 (9.1) [6.1-13.5] | 5 (5.7) [2.3-13.6] | 8 (25.3) [11.6-46.8] | 10 (8.1) [3.1-19.6] | 31 (6.4) [3.8-10.7] | 13 (7.9) [3.7-16.1] | 14 (25.9) [12.1-47.1] |
| 1 | 20 (31.6) [18.5-48.4] | 100 (59.7) [49.3-69.3] | 273 (49.1) [43.0-55.2] | 58 (67.2) [52.6-79.1] | 13 (41.8) [22.9-63.4] | 62 (51.3) [37.8-64.6] | 256 (53.0) [46.2-59.7] | 88 (51.9) [41.5-62.1] | 25 (44.9) [29.8-61.1] |
| 2-4 | 30 (49.3) [30.9-67.9] | 47 (28.2) [20.3-37.7] | 212 (38.1) [32.5-44.1] | 23 (26.2) [15.8-40.3] | 9 (28.9) [12.8-53.0] | 40 (33.4) [22.4-46.6] | 182 (37.5) [31.3-44.3] | 62 (36.6) [27.7-46.5] | 16 (28.5) [17.0-43.6] |
| ≥5 | 4 (7.0) [1.7-24.1] | 6 (3.3) [1.2-9.1] | 20 (3.7) [1.9-6.8] | 1 (0.9) [0.2-3.7] | 1 (4.0) [0.5-23.7] | 9 (7.2) [2.7-17.6] | 15 (3.0) [1.5-5.8] | 6 (3.7) [0.9-13.0] | 1 (0.7) [0.1-5.0] |
| Shotguns, No. | | | | | | | | | |
| 0 | 39 (62.3) [43.2-78.2] | 125 (75.0) [65.6-82-5] | 355 (63.8) [57.5-69.6] | 63 (73.0) [58.9-83.7] | 20 (65.4) [44.8-81.4] | 78 (64.4) [50.7-76.0] | 320 (66.2) [59.4-72.5] | 116 (68.3) [57.5-77.4] | 39 (70.7) [55.3-82.5] |
| 1 | 18 (28.5) [14.8-47.7] | 32 (19.3) [12.9-28.0] | 135 (24.3) [19.4-30.0] | 10 (11.9) [6.5-20.6] | 6 (20.4) [9.2-39.3] | 29 (24.0) [14.7-36.6] | 110 (22.8) [17.7-28.9] | 36 (21.2) [13.7-31.3] | 12 (22.3) [12.2-37.4] |
| 2-4 | 5 (8.1) [2.5-23.3] | 9 (5.6) [2.5-12.3] | 58 (10.4) [6.8-15.6] | 13 (15.1) [7.2-29.1] | 4 (14.2) [5.3-32.9] | 10 (8.4) [3.5-19.1] | 52 (10.8) [6.9-16.8] | 14 (8.2) [4.0-16.0] | 3 (5.4) [1.8-15.2] |
| ≥5 | 1 (1.1) [0.2-7.7] | 0 | 9 (1.6) [0.4-5.4] | 0 | 0 | 4 (3.2) [0.5-19.5] | 1 (0.1) [0.0-1.0] | 4 (2.3) [0.3-14.7] | 1 (1.6) [0.2-10.7] |
| Rifles, No. | | | | | | | | | |
| 0 | 42 (68.1) [48.7-82.8] | 129 (77.3) [68.3-84.3] | 374 (67.3) [61.3-72.8] | 56 (64.8) [48.2-78.5] | 17 (55.7) [34.3-75.3] | 87 (71.8) [58.5-82.0] | 324 (67.0) [60.3-73.1] | 128 (75.2) [65.6-82.9] | 37 (68.1) [52.5-80.5] |
| 1 | 15 (24.6) [11.9-44.0] | 29 (17.4) [11.4-25.8] | 130 (23.4) [18.6-29.0] | 12 (14.1) [7.1-26.1] | 11 (36.4) [18.4-59.2] | 22 (18.3) [10.5-29.8] | 111 (22.9) [17.6-29.2] | 28 (16.7) [10.6-25.3] | 11 (20.6) [11.3-34.6] |
| 2-4 | 4 (6.2) [1.4-23.7] | 7 (4.0) [1.7-9.3] | 44 (7.9) [5.2-11.7] | 18 (20.7) [9.7-38.7] | 1 (2.8) [0.5-13.8] | 12 (9.6) [3.8-22.1] | 46 (9.5) [6.2-14.4] | 8 (4.9) [2.5-9.3] | 5 (9.9) [3.6-24.3] |
| ≥5 | 1 (1.1) [0.2-7.7] | 2 (1.2) [0.3-5.5] | 8 (1.4) [0.5-4.2] | 1 (0.4) [0.1-2.7] | 2 (5.1) [1.0-22.0] | 1 (0.4) [0.1-2.9] | 3 (0.5) [0.2-1.8] | 5 (3.2) [0.7-13.4] | 1 (1.3) [0.2-8.9] |
| **Primary firearm reason** | | | | | | | | | |
| Gift or inheritance | 2 (3.3) [4.5-20.0] | 9 (5.5) [3.0-9.7] | 42 (7.8) [5.2-11.6] | 10 (12.2) [4.0-31.4] | 4 (12.6) [4.4-31.4] | 3 (2.4) [0.9-6.3] | 36 (7.5) [4.5-12.4] | 15 (9.0) [4.8-16.3] | 4 (7.3) [3.4-14.9] |

© 2024 Anestis MD et al. *JAMA Network Open.*

| Characteristic | Black adults, No. (%) [95% CI] | | | | | | | | |
|---|---|---|---|---|---|---|---|---|---|
| | Region | | | | Political beliefs | | | | |
| | Northeast | Midwest | South | West | Highly conservative | Somewhat conservative | Moderate | Somewhat liberal | Highly liberal |
| Safety at home | 33 (58.4) [38.4-75.9] | 110 (68.7) [58.1-77.7] | 349 (64.1) [58.1-69.7] | 51 (60.4) [43.7-74.9] | 16 (58.8) [35.7-78.5] | 81 (69.9) [55.2-81.4] | 306 (64.8) [57.9-71.1] | 108 (64.6) [54.9-73.2] | 25 (50.0) [34.9-65.0] |
| Safety away from home | 13 (23.0) [8.6-4.9] | 9 (5.8) [2.8-11.6] | 29 (5.3) [3.4-8.2] | 2 (2.5) [0.6-9.5] | 2 (4.3) [0.6-25.2] | 5 (4.2) [1.6-11.0] | 36 (7.6) [4.6-12.3] | 9 (5.4) [2.5-11.7] | 2 (4.5) [1.4-13.4] |
| Hunting | 0 | 2 (1.3) [0.2-8.9] | 15 (2.8) [1.4-5.5] | 1 (0.4) [0.1-2.5] | 1 (1.1) [0.1-7.4] | 6 (4.8) [1.1-18.8] | 5 (1.0) [0.3-2.8] | 2 (1.4) [0.5-3.6] | 4 (8.4) [2.2-27.6] |
| Other recreation | 3 (5.6) [1.4-19.2] | 2 (1.1) [0.3-3.3] | 13 (2.3) [1.1-4.6] | 6 (6.9) [2.0-20.9] | 2 (8.7) [1.3-41.7] | 1 (1.0) [0.2-4.3] | 15 (1.4) [0.5-3.6] | 2 (1.4) [0.5-3.6] | 2 (5.0) [1.5-15.3] |
| Belongs to someone else | 5 (8.8) [3.8-18.9] | 21 (13.4) [7.1-24.1] | 73 (13.4) [9.8-18.0] | 14 (16.9) [8.4-31.3] | 1 (2.9) [0.6-13.3] | 19 (16.6) [7.8-32.2] | 57 (12.1) [8.3-17.3] | 26 (15.3) [10.3-22.2] | 9 (19.2) [9.7-34.6] |
| **Firearm storage**[a] | | | | | | | | | |
| ≥1 Loaded | | | | | | | | | |
| Never | 16 (30.7) [17.2-48.8] | 47 (33.6) [23.3-45.8] | 189 (36.7) [29.6-42.2] | 49 (61.1) [44.1-75.8] | 10 (41.5) [20.8-65.7] | 38 (31.5) [19.2-47.1] | 168 (39.8) [32.8-47.2] | 59 (32.3) [23.4-42.6] | 22 (48.2) [32.6-64.2] |
| Rarely | 10 (16.5) [4.2-47.0] | 13 (7.7) [4.2-14.0] | 42 (8.8) [5.7-13.4] | 2 (2.4) [0.6-9.8] | 0 | 7 (5.8) [2.2-14.1] | 42 (10.0) [6.1-16.0] | 14 (9.1) [5.0-16.1] | 2 (3.9) [0.8-17.7] |
| Occasionally | 4 (6.7) [1.2-29.3] | 2 (1.7) [0.5-5.2] | 27 (5.7) [3.6-8.8] | 2 (2.5) [0.9-6.7] | 6 (24.6) [8.5-53.5] | 10 (9.5) [4.0-21.0] | 13 (3.0) [1.6-5.5] | 6 (3.8) [1.8-8.0] | 0 |
| Often | 1 (2.1) [0.5-8.4] | 6 (3.3) [1.3-8.0] | 12 (2.5) [1.1-5.4] | 7 (9.2) [1.8-3.6] | 1 (2.7) [0.5-14.1] | 1 (0.9) [0.1-6.4] | 15 (3.4) (1.3-8.4] | 9 (5.7) [2.0-15.1] | 1 (2.4) [0.5-10.0] |
| Almost always | 2 (3.0) [1.0-8.4] | 10 (7.5) [3.6-15.1] | 35 (7.3) [4.6-11.5] | 3 (2.8) [0.8-9.0] | 1 (3.5) [0.5-21.8] | 10 (9.1) [3.6-20.9] | 20 (4.9) [2.8-8.4] | 12 (8.0) [2.8-8.4] | 6 (14.1) [5.1-33.2] |
| Always | 22 (41.0) [24.1-60.4] | 66 (46.2) [33.9-58.9] | 199 (40.0) [33.8-46.7] | 18 (22.0) [12.3-36.2] | 7 (27.6) [10.4-55.7] | 46 (43.1) [29.0-58.5] | 171 (39.0) [32.1-46.3] | 61 (41.2) [30.4-52.9] | 14 (31.4) [19.3-46.7] |
| ≥1 With locking device | | | | | | | | | |
| Never | 12 (22.5) [11.0-40.4] | 54 (35.9) [24.8-48.8] | 177 (36.1) [29.9-42.7] | 37 (45.5) [29.4-62.6] | 6 (25.3) [9.6-51.9] | 34 (30.8) [18.6-46.5] | 163 (37.8) [30.7-45.3] | 55 (37.2) [27.0-48.6] | 15 (32.7) [19.4-49.7] |
| Rarely | 7 (12.5) [4.2-31.9] | 4 (3.3) [1.2-9.1] | 31 (5.9) [4.0-8.8] | 7 (9.1) [1.8-35.4] | 1 (2.3) [0.3-14.9] | 3 (2.5) [0.6-9.8] | 30 (6.9) [3.8-11.9] | 9 (6.2) [3.3-11.0] | 10 (14.3) [5.9-30.8] |
| Occasionally | 1 (1.6) [0.4-6.5] | 8 (6.0) [2.7-12.9] | 22 (4.3) [2.5-7.5] | 1 (1.2) [0.3-4.8] | 0 | 6 (5.6) [2.2-13.7] | 18 (4.0) [2.2-7.3] | 7 (5.0) [2.0-11.9] | 1 (0.9) [0.1-6.3] |
| Often | 1 (2.1) [0.5-8.4] | 5 (3.8) [1.4-9.7] | 17 (3.6) [2.0-6.4] | 2 (2.5) [0.8-7.7] | 2 (9.7) [2.0-36.1] | 5 (4.6) [1.7-11.9] | 13 (3.2) [1.7-11.9] | 4 (2.9) [1.1-7.2] | 1 (0.6) [0.1-4.2] |
| Almost always | 6 (12.1) [2.0-48.4] | 8 (5.5) [2.8-10.6] | 39 (7.9) [4.8-12.7] | 6 (7.2) [2.3-20.2] | 2 (6.5) [1.5-23.5] | 11 (10.2) [4.1-23.0] | 39 (9.2) [5.4-15.3] | 5 (3.5) [1.6-7.8] | 1 (3.1) [0.4-19.1] |

© 2024 Anestis MD et al. *JAMA Network Open.*

| Characteristic | Black adults, No. (%) [95% CI] | | | | | | | | |
|---|---|---|---|---|---|---|---|---|---|
| | Region | | | | Political beliefs | | | | |
| | Northeast | Midwest | South | West | Highly conservative | Somewhat conservative | Moderate | Somewhat liberal | Highly liberal |
| Always | 27 (49.3) [30.6-68.1] | 70 (45.5) [33.3-58.2] | 212 (42.1) [35.8-48.7] | 28 (34.6) [22.0-49.7] | 15 (56.3) [32.1-77.8] | 50 (46.2) [31.9-61.2] | 170 (39.0) [32.2-46.2] | 75 (45.3) [34.5-56.6] | 21 (48.3) [32.8-64.2] |
| ≥1 In locked location | | | | | | | | | |
| Never | 9 (16.9) [7.3-34.5] | 40 (26.0) [16.9-37.7] | 143 (29.0) [23.4-35.2] | 31 (37.6) [22.2-56.0] | 8 (30.2) [12.9-55.8] | 23 (21.2) [11.0-36.7] | 127 (29.2) [22.8-36.6] | 47 (31.0) [22.2-41.4] | 13 (28.4) [16.8-43.8] |
| Rarely | 3 (5.8) [1.4-8.1] | 5 (3.5) [1.4-8.1] | 32 (6.3) [4.1-9.4] | 7 (9.1) [1.8-35.4] | 0 | 5 (4.6) [1.7-11.7] | 27 (6.7) [3.8-11.5] | 10 (7.1) [3.4-13.9] | 3 (2.4) [0.8-7.5] |
| Occasionally | 1 (2.5) [0.8-8.0] | 10 (6.9) [3.0-15.3] | 16 (3.3) [1.6-7.0] | 5 (6.0) [2.5-13.5] | 2 (7.4) [1.0-38.0] | 1 (0.8) [0.2-3.3] | 23 (5.5) [3.0-9.8] | 6 (3.5) [1.4-8.3] | 1 (1.5) [0.2-9.9] |
| Often | 2 (3.6) [1.3-10.1] | 5 (4.0) [1.4-10.7] | 20 (3.6) [2.0-6.5] | 5 (5.8) [1.3-22.4] | 1 (2.3) [0.3-15.2] | 7 (7.0) [2.3-19.6] | 17 (3.3) [1.7-6.3] | 6 (4.4) [1.7-11.1] | 1 (0.4) [0.1-2.9] |
| Almost always | 8 (15.4) [3.6-46.6] | 6 (3.6) [1.7-7.5] | 56 (11.7) [7.6-17.7] | 2 (2.3) [0.5-10.1] | 4 (17.5) [4.8-47.1] | 11 (10.0) [4.8-19.7] | 42 (10.3) [6.0-17.3] | 14 (8.6) [3.6-19.3] | 1 (0.8) [0.1-5.7] |
| Always | 30 (55.9) [35.9-74.1] | 81 (56.1) [43.8-67.6] | 228 (46.1) [39.7-527] | 32 (39.2) [25.4-54.9] | 12 (42.7) [21.2-67.2] | 61 (56.4) [41.5-70.2] | 189 (45.0) [37.0-52.3] | 74 (45.5) [34.6-56.8] | 30 (66.5) [51.1-79.0] |
| ≥1 In vehicle unlocked | | | | | | | | | |
| Never | 41 (75.5) [56.0-88.2] | 126 (85.5) [75.3-92.0] | 60 (70.1) [63.8-75.7] | 67 (81.5) [62.0-92.2] | 15 (52.3) [28.4-75.2] | 70 (61.6) [46.6-74.6] | 329 (76.8) [70.0-82.4] | 131 (78.9) [68.6-86.5] | 36 (78.2) [61.2-89.1] |
| Rarely | 2 (3.4) [1.1-10.0] | 8 (4.3) [2.0-8.9] | 49 (10.4) [6.9-15.3] | 5 (6.1) [2.0-17.0] | 3 (11.5) [1.9-47.2] | 13 (12.1) [5.8-23.5] | 38 (8.9) [5.4-14.1] | 7 (4.9) [2.5-9.4] | 2 (5.5) [1.8-16.1] |
| Occasionally | 0 | 9 (6.3) [1.9-18.8] | 24 (4.9) [2.9-8.2] | 3 (4.1) [1.4-11.9] | 2 (7.4) [1.0-38.0] | 7 (5.7) [1.9-15.9] | 20 (4.8) [2.5-9.0] | 6 (4.5) [1.6-12.0] | 1 (0.8) [0.1-5.3] |
| Often | 1 (2.1) [0.5-8.4] | 4 (1.6) [0.5-5.2] | 15 (3.0) [1.4-6.4] | 1 (0.1) [0.0-1.0] | 1 (2.3) [0.3-15.2] | 6 (5.7) [1.5-20.1] | 10 (2.1) [1.0-4.5] | 3 (1.7) [0.4-6.6] | 0 |
| Almost always | 2 (3.9) [0.5-23.1] | 1 (0.1) [0.0-0.1] | 16 (3.5) [1.7-7.1] | 0 | 0 | 2 (1.8) [0.3-10.4] | 10 (2.3) [0.9-5.6] | 7 (4.9) [1.5-14.9] | 0 |
| Always | 8 (15.1) [5.3-36.0] | 4 (2.3) [0.8-5.5] | 40 (8.1) [5.3-12.2] | 6 (7.7) [1.1-37.9] | 6 (26.5) [9.7-54.6] | 14 (13.1) [5.7-27.3] | 23 (5.1) [2.6-9.9] | 9 (5.1) [1.8-13.5] | 7 (15.5) [6.3-33.5] |
| **Firearm carrying** | | | | | | | | | |
| Frequency | | | | | | | | | |
| Never | 26 (45.7) [28.4-64.1] | 87 (54.7) [43.5-65.6] | 278 (50.6) [44.5-56.8] | 62 (72.8) [55.8-85.1] | 8 (27.1) [13.9-46.1] | 51 (43.9) [30.5-58.2] | 248 (51.8) [45.0-58.6] | 103 (61.5) [50.7-71.3] | 35 (71.8) [567-83.2] |
| Rarely | 2 (2.9) [0.6-13.6] | 27 (16.8) [10.1-26.6] | 94 (17.2) [13.0-22.3] | 11 (13.1) [6.3-25.2] | 9 (31.5) [14.3-56.0] | 25 (21.9) [12.2-36.1] | 71 (14.8) [10.7-20.2] | 23 (13.5) [8.5-20.9] | 6 (11.3) [4.5-25.7] |

© 2024 Anestis MD et al. *JAMA Network Open.*

| Characteristic | Black adults, No. (%) [95% CI] | | | | | | | | |
|---|---|---|---|---|---|---|---|---|---|
| | Region | | | | Political beliefs | | | | |
| | Northeast | Midwest | South | West | Highly conservative | Somewhat conservative | Moderate | Somewhat liberal | Highly liberal |
| Sometimes | 6 (10.9) [3.7-27.9] | 13 (7.9) [4.4-13.8] | 74 (13.4) [9.9-18.0] | 2 (2.4) [0.7-7.3] | 8 (26.8) [11.7-50.2] | 15 (12.8) [6.7-23.0] | 49 (10.3) [7.0-14.9] | 19 (11.4) [6.4-19.5] | 4 (8.0) [2.9-20.1] |
| Frequently | 4 (6.9) [1.5-26.6] | 7 (4.6) [1.9-10.9] | 28 (5.1) [3.1-8.2] | 1 (1.1) [0.3-3.7] | 1 (0.3) [0.0-2.3] | 7 (6.3) [2.3-16.2] | 26 (5.4) [3.2-8.8] | 2 (1.4) [0.4-4.8] | 2 (4.2) [1.0-15.6] |
| Almost always | 11 (19.9) [6.4-47.3] | 17 (11.0) [4.8-23.1] | 23 (4.2) [2.7-6.6] | 8(9.5) [2.2-33.4] | 1 (2.9) [0.4-18.6] | 5 (4.5) [1.2-14.9] | 41 (8.6) [5.2-13.9] | 11 (6.3) [1.9-18.9] | 2 (4.7) [1.5-13.8] |
| Always | 8 (13.7) [4.7-34.2] | 8 (5.0) [2.5-9.7] | 52 (9.5) [6.1-14.5] | 1 (1.1) [0.2-7.8] | 3 (11.3) [1.9-45.1] | 12 (10.6) [4.1-24.9] | 44 (9.1) [5.8-14.1] | 10 (5.7) [2.2-14.1] | 0 |
| Reason | | | | | | | | | |
| Lack of faith in police | 9 (28.0) [7.1-66.4] | 6 (9.0) [3.7-20.0] | 43 (15.5) [10.1-22.9] | 3 (10.4) [3.0-30.7] | 3 (16.3) [3.5-51.0] | 6 (8.8) [3.4-20.8] | 38 (15.7) [9.6-24.7] | 13 (20.3) [8.9-40.1] | 2 (8.8) [1.2-42.9] |
| Required for job | 6 (20.2) [5.3-53.3] | 1 (1.7) [0.3-8.0] | 22 (8.1) [4.5-14.0] | 1 (2.4) [0.5-10.9] | 2 (11.9) [2.5-41.0] | 6 (8.6) [2.5-25.8] | 16 (6.9) [3.6-12.9] | 6 (9.1) [2.3-30.2] | 0 |
| Expression of freedom | 9 (30.4) [8.6-66.8] | 8 (11.5) [4.5-26.6] | 17 (6.3) [3.4-11.5] | 2 (6.7) [1.1-32.2] | 4 (19.9) [7.0-44.8] | 7 (11.0) [3.6-28.8] | 23 (9.9) [4.9-18.8] | 1 (2.3) [0.5-10.0] | 1 (6.2) [0.8-34.1] |
| Self-protection | 30 (98.4) [88.4-99.8] | 66 (92.3) [81.2-97.1] | 235 (86.7) [78.6-92.1] | 19 (80.3) [49.5-94.4] | 4 (78.9) [45.5-94.4] | 6 (90.2) [73.5-96.8] | 26 (88.5) [80.0-93.7] | 9 (85.3) [66.9-94.3] | 14 (100) |
| Protect others | 16 (52.3) [24.6-78.7] | 29 (41.0) [26.7-57.0] | 89 (33.1) [25.5-41.7] | 5 (20.9) [6.3-51.0] | 9 (43.5) [19.7-70.6] | 23 (34.8) [19.5-54.0] | 85 (37.3) [28.4-47.2] | 18 (27.7) [15.3-44.8] | 5 (36.8) [15.7-64.6] |
| Hunting or recreation | 2 (7.2) [1.6-27.0] | 2 (3.1) [0.8-10.6] | 22 (8.1) [4.6-13.8] | 5 (21.0) [5.4-55.4] | 7 (35.5) [13.7-65.6] | 7 (11.0) [3.5-29.8] | 12 (5.2) [2.5-10.5] | 3 (4.5) [1.7-11.5] | 2 (13.2) [3.1-41.7] |
| Location | | | | | | | | | |
| Vehicle | 25 (83.0) [54.6-95.2] | 54 (75.3) [60.8-85.7] | 234 (86.6) [80.9-90.8] | 20 (89.3) [70.7-96.7] | 16 (77.4) [47.9-92.7] | 51 (78.7) [61.9-89.3] | 200 (87.6) [81.8-91.7] | 51 (79.9) [64.2-89.9] | 13 (93.4) [64.6-99.1] |
| Walking on the street | 27 (88.1) [70.0-95.9] | 41 (56.5) [40.3-71.4] | 112 (41.4) [33.0-50.2] | 11 (47.9) [19.2-78.1] | 9 (44.2) [19.5-72.1] | 26 (40.4) [23.6-59.8] | 120 (52.7) [42.9-62.2] | 26 (39.8) [23.9-58.2] | 5 (40.2) [18.1-67.3] |
| Retail stores | 23 (75.1) [50.0-90.1] | 24 (32.9) [18.8-51.1] | 60 (22.1) [16.1-29.5] | 3 (12.7) [3.8-34.6] | 5 (26.1) [8.4-57.5] | 12 (18.2) [8.0-36.2] | 71 (30.9) [22.8-40.3] | 18 (27.6) [12.6-50.3] | 4 (26.6) [10.0-54.3] |
| Restaurants | 23 (75.1) [50.0-90.1] | 23 (32.6) [18.5-50.8] | 49 (18.1) [13.0-24.7] | 3 (11.2) [3.1-33.3] | 1 (4.7) [0.9-20.1] | 11 (17.5) [7.5-35.5] | 67 (29.4) [21.5-38.7] | 16 (24.2) [10.6-46.4] | 3 (20.4) [6.6-48.2] |
| Places of worship | 6 (18.7) [4.9-50.7] | 6 (7.9) [3.3-17.7] | 30 (11.0) [7.1-16.7] | 2 (7.8) [1.5-32.6] | 3 (13.2) [3.9-36.6] | 8 (11.9) [4.7-26.9] | 26 (11.2) [6.8-17.9] | 6 (9.4) [3.4-23.6] | 1 (5.0) [0.7-29.3] |
| Parks | 15 (50.6) [23.1-77.8] | 18 (24.8) [12.0-44.4] | 68 (25.1) [18.3-33.4] | 2 (9.5) [2.2-32.2] | 3 (15.8) [3.3-51.2] | 15 (23.2) [11.0-42.4] | 59 (25.8) [18.4-34.9] | 23 (35.1) [19.1-55.2] | 3 (25.4) [9.1-53.4] |
| Others’ homes | 16 (51.3) [23.8-78.0] | 18 (25.6) [12.7-44.8] | 31 (11.6) [7.7-17.1] | 2 (6.9) [1.1-32.8] | 1 (3.4) [0.4-21.4] | 5 (8.3) [2.4-25.1] | 44 (19.1) [12.5-27.9] | 15 (23.7) [10.4-45.4] | 2 (15.1) [3.9-43.6] |

© 2024 Anestis MD et al. *JAMA Network Open.*

| Characteristic | Black adults, No. (%) [95% CI] | | | | | | | | |
|---|---|---|---|---|---|---|---|---|---|
| | Region | | | | Political beliefs | | | | |
| | Northeast | Midwest | South | West | Highly conservative | Somewhat conservative | Moderate | Somewhat liberal | Highly liberal |
| **Firearm violence exposure** | | | | | | | | | |
| Threatened with firearm | 113 (22.3) [17.1-28.5] | 123 (26.0) [21.0-31.8] | 356 (20.9) [18.3-23.9] | 57 (18.2) [13.8-23.4] | 30 (24.8) [15.1-38.1] | 75 (21.1) [15.8-27.7] | 357 (22.1) [19.3-25.2] | 118 (20.6) [16.4-25.6] | 59 (21.1) [15.0-28.9] |
| Shot | 17 (3.4) [1.6-7.0] | 9 (1.9) [0.9-4.0] | 51 (3.0) [2.1-4.4] | 3 (1.0) [0.5-2.2] | 3 (2.3) [0.8-6.7] | 4 (1.1) [0.5-2.5] | 49 (3.1) [2.1-4.5] | 15 (2.6) [1.2-5.7] | 9 (3.1) [1.3-7.2] |
| Friend or family member shot | 207 (40.6) [34.1-47.4] | 240 (50.7) [44.5-56.9] | 670 (39.6) [36.2-43.0] | 12 (38.0) [30.9-45.6] | 47 (39.2) [27.8-52.1] | 132 (38.2) [31.4-45.6] | 673 (41.6) [38.2-45.2] | 247 (42.9) [37.2-48.9] | 119 (42.4) [34.3-51.0] |
| Shooting in neighborhood | 203 (39.7) [33.3-46.5] | 215 (45.4) [39.3-51.6] | 602 (35.8) [32.5-39.2] | 118 (37.5) [30.4-45.3] | 40 (33.5) [22.6-46.5] | 124 (36.7) [29.6-44.5] | 618 (38.4) [34.9-41.9] | 227 (39.2) [33.6-45.1] | 107 (38.1) [30.4-46.4] |

[a]Rarely, 1% to 25% of the time; occasionally, 26% to 50%; often, 51% to 75%; almost always, 76% to 99%.

© 2024 Anestis MD et al. *JAMA Network Open.*

**eTable 3.** Sex, Age, and Metropolitan Area Differences in Firearm Access, Behaviors, and Exposure Among American Indian or Alaska Native Adults

| Characteristic | American Indian or Alaska Native adults, No. (%) [95% CI] | | | | | | | | |
|---|---|---|---|---|---|---|---|---|---|
| | Total | Sex | | Age, y | | | | Metropolitan area status | |
| | | Female | Male | 18-29 | 30-44 | 45-59 | ≥60 | Nonmetropolitan | Metropolitan |
| **Firearm in or around home** | | | | | | | | | |
| Any | 238 (45.5) [39.4-51.7] | 105 (37.8) [30.3-45.9] | 133 (54.2) [44.6-63.5] | 24 (29.1) [15.2-48.4] | 81 (41.9) [31.7-52.8] | 62 (50.1) [38.1-62.1] | 71 (57.4) [47.6-66.7] | 76 (58.7) [44.7-71.3] | 162 (41.1) [34.6-47.9] |
| Handguns, No. | | | | | | | | | |
| 0 | 29 (14.0) [9.1-21.0] | 13 (14.5) [8.8-23.0] | 16 (13.6) [6.9-25.2] | 4 (23.7) [7.4-54.9] | 9 (12.3) [4.4-30.0] | 7 (14.1) [6.6-27.7] | 9 (13.7) [7.7-23.3] | 9 (14.8) [5.9-32.8] | 20 (13.7) [8.5-21.2] |
| 1 | 80 (38.0) [30.2-46.6] | 35 (38.0) [27.2-50.2] | 45 (38.0) [27.3-50.1] | 6 (37.3) [15.1-66.4] | 28 (37.0) [22.9-53.8] | 20 (37.9) [23.7-54.4] | 26 (39.5) [27.8-52.5] | 17 (27.5) [15.2-44.5] | 62 (42.5) [33.3-52.4] |
| 2-4 | 84 (40.1) [31.7-49.2] | 36 (39.4) [27.5-52.8] | 48 (40.6) [29.4-53.0] | 6 (39.0) [14.7-70.4] | 35 (47.3) [31.2-63.9] | 22 (41.8) [26.4-58.9] | 20 (30.8) [20.2-44.0] | 29 (46.5) [29.5-64.4] | 54 (37.4) [28.3-47.4] |
| ≥5 | 16 (7.8) [4.2-14.2] | 7 (8.0) [2.5-23.2] | 9 (7.7) [4.1-14.1] | 0 | 3 (3.4) [0.7-15.2] | 3 (6.3) [2.6-14.3] | 10 (16.0) [7.0-32.5] | 7 (11.2) [5.1-22.7] | 9 (6.4) [2.5-15.7] |
| Shotguns, No. | | | | | | | | | |
| 0 | 81 (39.0) [31.3-47.4] | 43 (47.4) [35.3-59.8] | 38 (32.5) [23.0-43.8] | 10 (64.1) [31.1-87.6] | 17 (22.6) [13.0-36.4] | 26 (49.3) [33.4-65.3] | 29 (43.8) (31.5-57.0] | 18 (29.0) [17.1-44.9] | 63 (43.3) [34.1-53.0] |
| 1 | 73 (35.3) [27.0-44.5] | 22 (24.3) [14.9-37.0] | 51 (43.8) [32.0-56.2] | 1 (9.5) [1.3-45.6] | 36 (48.8) [32.6-65.3] | 12 (22.5) [11.7-39.0] | 24 (36.0) [23.8-50.2] | 21 (34.4) [18.7-54.4] | 52 (35.6) [26.5-45.9] |
| 2-4 | 47 (22.4) [15.8-30.9] | 23 (25.2) [14.9-39.4] | 24 (20.3) [12.5-31.1] | 4 (26.3) [6.7-63.9] | 19 (25.3) [13.3-42.9] | 14 (26.0) [13.7-43.9] | 10 (15.3) [9.0-24.8] | 20 (31.4) [17.3-50.1] | 27 (18.6) [11.9-27.8] |
| ≥5 | 7 (3.3) [1.4-7.5] | 3 (3.1) [0.7-12.2] | 4 (3.4) [1.2-9.5] | 0 | 2 (3.2) [0.6-15.2] | 1 (2.2) [0.6-7.3] | 3 (4.9) [1.4-15.9] | 3 (5.1) [1.4-16.9] | 4 (2.5) [0.8-7.7] |
| Rifles, No. | | | | | | | | | |
| 0 | 72 (34.8) [27.1-43.3] | 40 (44.5) [32.5-57.2] | 32 (27.2) [18.3-38.5] | 10 (65.3) [31.9-88.3] | 24 (31.8) [18.5-49.0] | 19 (36.6) [22.7-53.0] | 19 (29.8) [19.6-42.4] | 13 (21.2) [9.9-39.7] | 59 (40.6) [31.5-50.4] |
| 1 | 62 (29.5) [22.1-38.1] | 23 (25.1) [16.4-36.3] | 39 (32.9) [22.3-45.6] | 1 (8.4) [1.1-42.3] | 24 (31.9) [18.2-49.6] | 14 (27.0) [14.6-44.5] | 22 (33.6) [22.8-46.3] | 23 (37.5) [21.8-56.4] | 38 (26.1) [18.7-35.1] |
| 2-4 | 51 (24.7) [17.8-33.3] | 17 (19.0) [10.4-32.2] | 34 (29.2) [19.5-41.2] | 1 (8.9) [1.2-43.7] | 21 (28.1) [15.5-45.6] | 10 (19.1) [9.9-33.7] | 19 (29.0) [17.6-44.0] | 18 (28.7) [15.8-46.5] | 34 (23.0) [15.3-33.1] |
| ≥5 | 22 (11.0) [6.6-17.9] | 10 (11.4) [5.2-23.2] | 12 (10.6) [5.3-20.2] | 3 (17.5) [2.6-62.6] | 6 (8.2) [3.2-19.6] | 9 (17.3) [7.4-35.4] | 5 (7.6) [3.0-18.2] | 8 (12.6) [5.5-26.3] | 15 (10.3) [5.3-19.1] |
| **Primary firearm reason** | | | | | | | | | |
| Gift or inheritance | 14 (7.1) [4.5-11.0] | 5 (5.6) [2.5-12.1] | 9 (8.3) [4.8-14.1] | 0 | 5 (7.3) [3.1-16.2] | 2 (3.2) [1.0-9.5] | 7 (11.4) [6.4-19.6] | 3 (4.6) [1.6-12.5] | 11 (8.2) [5.0-13.3] |

© 2024 Anestis MD et al. *JAMA Network Open.*

| Characteristic | American Indian or Alaska Native adults, No. (%) [95% CI] | | | | | | | | |
|---|---|---|---|---|---|---|---|---|---|
| | Total | Sex | | Age, y | | | | Metropolitan area status | |
| | | Female | Male | 18-29 | 30-44 | 45-59 | ≥60 | Nonmetropolitan | Metropolitan |
| Safety at home | 115 (57.1) [48.3-65.5] | 46 (51.6) [38.9-64.1] | 69 (61.5) [49.4-72.3] | 8 (57.9) [28.1-82.9] | 42 (55.6) [38.9-71.2] | 33 (68.3) [51.1-81.7] | 32 (50.2) [37.2-63.3] | 37 (60.2) [41.6-76.4] | 78 (55.7) [45.8-65.2] |
| Safety away from home | 15 (7.2) [3.4-14.6] | 9 (9.8) [3.3-25.5] | 6 (5.2) [2.1-12.5] | 0 | 7 (9.7) [2.6-30.0] | 3 (6.2) [1.9-18.2] | 4 (6.7) [2.6-16.5] | 6 (9.1) [1.9-34.1] | 9 (6.4) [3.1-12.7] |
| Hunting | 16 (7.8) [4.1-14.4] | 3 (3.6) [1.1-11.3] | 13 (11.1) [5.3-22.1] | 0 | 8 (10.6) [3.4-28.2] | 3 (6.1) [1.9-18.1] | 5 (7.6) [3.5-16.0] | 7 (10.8) [3.0-32.0] | 9 (6.5) [3.4-11.9] |
| Other recreation | 4 (2.0) [0.9-4.7] | 1 (1.4) [0.3-6.0] | 3 (2.5) [0.9-7.0] | 0 | 1 (1.1) [0.2-7.9] | 1 (2.2) [0.3-14.1] | 2 (3.4) [1.2-9.1] | 1 (1.8) [0.4-8.5] | 3 (2.1) [0.8-5.7] |
| Belongs to someone else | 26 (12.9) [8.5-19.2] | 19 (21.6) [13.5-32.7] | 7 (6.1) [2.5-14.4] | 6 (42.1) [17.1-71.9] | 9 (12.2) [5.3-25.7] | 3 (6.2) [2.3-15.5] | 8 (12.4) [6.2-23.2] | 4 (7.1) [2.7-17.3] | 22 (15.5) [9.8-23.8] |
| **Firearm storage**[a] | | | | | | | | | |
| ≥1 Loaded | | | | | | | | | |
| Never | 85 (43.5) [34.9-52.5] | 46 (56.3) [43.3-68.5] | 39 (34.8) [24.3-47.0] | 8 (54.4) [25.4-80.7] | 31 (45.7) [29.3-63.1] | 14 (31.5) [18.7-47.9] | 31 (47.5) [34.5-60.8] | 24 (42.7) [26.4-60.8] | 61 (43.8) [34.1-54.1] |
| Rarely | 10 (5.4) [3.1-9.2] | 7 (9.1) [4.6-17.3] | 3 (2.9) [1.1-7.1] | 1 (4.9) [0.6-29.2] | 5 (8.1) [3.4-18.3] | 2 (4.6) [1.5-13.2] | 2 (3.3) [1.3-8.3] | 3 (5.7) [2.2-14.1] | 7 (5.3) [2.7-10.2] |
| Occasionally | 14 (7.5) [3.7-14.7] | 4 (5.3) [2.1-12.6] | 10 (9.0) [3.5-21.0] | 1 (9.4) [1.3-45.6] | 9 (13.6) [4.9-32.5] | 1 (1.6) [0.2-10.8] | 3 (5.0) [1.8-13.0] | 5 (9.1) [2.2-31.5] | 9 (6.8) [3.1-14.0] |
| Often | 3 (1.7) [0.5-5.7] | 2 (2.2) [0.3-12.8] | 1 (1.3) [0.3-6.5] | 0 | 2 (2.4) [0.3-15.5] | 1 (0.2) [0.0-1.4] | 1 (2.4) [0.5-11.0] | 0 | 3 (2.4) [0.7-7.9] |
| Almost always | 10 (4.8) [2.2-10.0] | 3 (2.4) [0.5-10.7] | 7 (6.4) [2.7-14.6] | 2 (13.8) [2.9-46.4] | 4 (4.1) [0.6-24.3] | 1 (3.0) [0.9-9.9] | 3 (4.7) [1.8-11.6] | 0 | 10 (6.8) [3.2-13.9] |
| Always | 76 (37.2) [28.6-46.5] | 23 (24.7) [14.7-38.4] | 53 (45.7) [33.7-58.1] | 2 (17.5) [4.2-50.7] | 19 (26.0) [13.0-45.1] | 31 (59.1) [42.4-73.9] | 23 (37.1) [24.8-51.5] | 25 (42.5) [26.1-60.7] | 51 (34.9) [25.3-45.8] |
| ≥1 With locking device | | | | | | | | | |
| Never | 80 (40.0) [31.6-49.1] | 3 (35.1) [24.2-47.7] | 49 (43.4) [31.8-55.8] | 5 (34.6) [12.7-65.8] | 26 (39.5) [24.2-57.2] | 17 (29.4) [15.6-48.3] | 31 (49.6) [36.4-62.8] | 26 (43.0) [26.6-61.1] | 53 (38.8) [29.5-49.0] |
| Rarely | 10 (5.4) [2.8-10.1] | 5 (6.7) [2.8-15.4] | 5 (4.4) [1.6-11.3] | 0 | 4 (6.4) [2.1-17.5] | 3 (7.4) [2.2-22.1] | 3 (4.0) [1.3-11.4] | 3 (5.3) [1.5-17.2] | 7 (5.4) [2.5-11.3] |
| Occasionally | 7 (3.5) [1.4-8.7] | 2 (2.8) [0.8-9.6] | 5 (4.1) [1.2-12.9] | 0 | 4 (5.6) [1.2-22.5] | 1 (2.4) [0.7-8.2] | 2 (3.1) [1.0-9.5] | 1 (1.8) [0.2-11.9] | 6 (4.3) [1.5-11.4] |
| Often | 7 (3.7) [1.2-11.4] | 2 (2.3) [0.8-6.6] | 5 (4.7) [1.0-19.0] | 0 | 4 (6.2) [0.9-32.9] | 2 (3.3) [1.0-10.7] | 1 (2.4) [0.8-7.0] | 1 (1.2) [0.2-8.4] | 6 (4.8) [1.3-15.7] |
| Almost always | 13 (7.0) [3.2-14.8] | 5 (7.1) [1.7-25.4] | 8 (7.0) [2.9-16.2] | 1 (4.9) [0.6-29.2] | 5 (7.3) [1.8-25.3] | 2 (3.5) [1.2-10.1] | 6 (9.8) [2.8-29.0] | 4 (6.6) [1.9-20.3] | 10 (7.3) [2.7-18.0] |

© 2024 Anestis MD et al. *JAMA Network Open.*

| Characteristic | American Indian or Alaska Native adults, No. (%) [95% CI] | | | | | | | | |
|---|---|---|---|---|---|---|---|---|---|
| | Total | Sex | | Age, y | | | | Metropolitan area status | |
| | | Female | Male | 18-29 | 30-44 | 45-59 | ≥60 | Nonmetropolitan | Metropolitan |
| Always | 77 (40.3) [31.8-49.4] | 36 (45.9) [33.5-58.9] | 41 (36.4) [25.5-48.9] | 9 (60.5) [30.4-84.4] | 23 (35.1) [20.6-53.0] | 25 (54.1) [36.8-70.4] | 21 (31.1) [20.4-44.3] | 23 (42.1) [25.6-60.6] | 54 (39.5) [30.1-49.7] |
| ≥1 In locked location | | | | | | | | | |
| Never | 48 (23.5) [17.1-31.3] | 28 (31.9) [21.6-44.3] | 20 (17.7) [10.5-28.2] | 4 (28.3) [9.1-60.9] | 12 (16.7) [7.4-33.2] | 9 (15.5) [7.4-33.2] | 22 (35.1) [24.0-48.0] | 15 (27.4) [14.9-44.8] | 32 (21.8) [15.1-30.5] |
| Rarely | 10 (5.0) [2.1-11.6] | 4 (4.8) [1.7-12.8] | 6 (5.1) [1.4-17.0] | 0 | 6 (8.5) [2.1-28.5] | 2 (4.1) [2.1-28.5] | 2 (3.2) [0.9-10.8] | 4 (7.5) [1.4-32.4] | 5 (3.9) [1.7-8.6] |
| Occasionally | 4 (2.6) [0.8-7.6] | 1 (1.9) [0.4-8.1] | 3 (3.0) [0.7-12.2] | 0 | 2 (3.7) [0.5-22.2] | 1 (3.1) [0.9-10.4] | 1 (1.6) [0.2-10.5] | 1 (1.8) [0.2-11.9] | 4 (2.9) [0.8-10.2] |
| Often | 7 (2.3) [1.0-5.0] | 3 (3.3) [1.1-9.8] | 4 (1.6) [0.5-4.5] | 0 | 0 | 2 (4.0) [1.0-14.6] | 2 (3.9) [1.5-9.7] | 1 (1.9) [0.4-7.9] | 5 (2.5) [1.0-6.2] |
| Almost always | 16 (8.3) [4.5-14.6] | 5 (6.2) [2.7-13.7] | 11 (9.7) [4.4-20.0] | 1 (3.7) [0.5-23.3] | 8 (12.4) [4.7-29.0] | 3 (7.4) [2.7-18.7] | 4 (5.8) [2.0-15.6] | 2 (3.2) [0.4-19.8] | 14 (10.4) [5.6-18.7] |
| Always | 117 (58.4) [49.5-66.8] | 46 (51.8) [39.1-64.3] | 71 (62.9) [50.5-73.8] | 10 (68.1) [36.6-88.7] | 45 (58.8) [40.9-74.6] | 30 (65.9) [49.5-79.3] | 32 (50.5) [37.3-63.6] | 37 (58.3) [40.2-74.4] | 80 (58.5) [48.3-68.0] |
| ≥1 In vehicle unlocked | | | | | | | | | |
| Never | 139 (70.3) [60.7-78.4] | 62 (72.8) [58.7-83.4] | 77 (68.6) [55.3-79.4] | 10 (73.2) [40.9-91.5] | 46 (64.8) [46.1-79.9] | 37 (76.9) [57.8-89.0] | 46 (70.5) [54.7-82.5] | 40 (67.7) [49.0-82.1] | 99 (71.4) [60.0-80.6] |
| Rarely | 21 (10.9) [6.1-18.7] | 9 (11.3) [4.2-26.7] | 12 (10.7) [5.2-20.5] | 0 | 8 (12.2) [4.9-27.5] | 5 (10.7) [3.9-26.2] | 8 (12.1) [4.2-30.2] | 2 (4.0) [1.4-10.9] | 18 (13.8) [7.4-24.4] |
| Occasionally | 7 (3.7) [1.1-11.6] | 2 (2.6) [0.8-8.1] | 5 (4.4) [0.9-19.6] | 0 | 4 (6.9) [1.2-31.3] | 1 (0.4) [0.1-3.1] | 2 (3.6) [1.1-11.6] | 1 (2.4) [0.5-10.8] | 6 (4.2) [1.0-16.2] |
| Often | 4 (2.1) [0.8-5.0] | 2 (2.9) [0.9-8.9] | 2 (1.5) [0.3-6.2] | 1 (7.7) [1.0-40.3] | 2 (2.5) [0.6-9.7] | 1 (1.4) [0.2-9.3] | 1 (0.9) [0.1-6.0] | 3 (5.9) [2.1-15.7] | 1 (0.4) [0.1-2.9] |
| Almost always | 9 (2.3) [0.8-6.6] | 5 (0.6) [0.1-4.3] | 4 (3.4) [1.0-10.8] | 0 | 1 (0.7) [0.1-5.1] | 0 | 4 (6.1) [1.9-18.1] | 6 (3.2) [0.4-19.8] | 3 (1.9) [0.6-6.3] |
| Always | 20 (10.7) [5.7-19.3] | 7 (9.8) [4.0-21.9] | 13 (11.4) [4.8-24.8] | 3 (19.0) [4.7-53.1] | 8 (12.9) [4.3-32.8] | 5 (10.6) [2.8-32.9] | 4 (6.8) [1.9-21.6] | 9 (16.7) [6.0-38.7] | 11 (8.2) [3.7-17.1] |
| **Firearm carrying** | | | | | | | | | |
| Frequency | | | | | | | | | |
| Never | 80 (39.2) [31.2-47.9] | 49 (55.1) [41.9-67.6] | 31 (26.9) [17.7-38.6] | 7 (49.4) [21.5-77.6] | 24 (32.7) [19.6-49.5] | 20 (39.8) [25.2-56.5] | 28 (44.1) [31.6-57.3] | 18 (28.9) [16.1-46.1] | 62 (43.8) [34.3-53.8] |
| Rarely | 48 (23.9) [16.9-32.6] | 19 (21.7) [12.3-35.4] | 29 (25.6) [16.3-37.7] | 5 (33.1) [10.4-67.9] | 19 (25.8) [13.5-43.6] | 7 (13.7) [6.4-27.0] | 18 (27.5) [16.6-42.0] | 12 (18.9) [8.8-36.0] | 37 (26.1) [17.7-36.6] |

© 2024 Anestis MD et al. *JAMA Network Open.*

| Characteristic | American Indian or Alaska Native adults, No. (%) [95% CI] | | | | | | | | |
|---|---|---|---|---|---|---|---|---|---|
| | Total | Sex | | Age, y | | | | Metropolitan area status | |
| | | Female | Male | 18-29 | 30-44 | 45-59 | ≥60 | Nonmetropolitan | Metropolitan |
| Sometimes | 16 (8.0) [4.4-14.1] | 5 (5.8) [2.4-13.3] | 11 (9.7) [4.5-19.9] | 0 | 5 (6.8) [2.9-15.3] | 4 (8.1) [1.6-31.9] | 7 (11.1) [4.9-23.3] | 6 (9.2) [3.4-22.5] | 11 (7.5) [3.5-15.3] |
| Frequently | 21 (10.0) [5.8-16.5] | 5 (5.1) [1.5-15.7] | 16 (13.8) [7.5-23.8] | 1 (7.7) [1.0-40.2] | 8 (11.0) [4.1-26.7] | 7 (13.6) [5.6-29.5] | 4 (6.4) [2.8-14.3] | 8 (13.2) [5.3-29.3] | 12 (8.6) [4.4-16.0] |
| Almost always | 20 (9.7) [5.3-17.2] | 7 (7.8) [2.1-24.5] | 13 (11.2) [5.9-20.5] | 0 | 11 (15.0) [5.9-33.3] | 5 (10.1) [3.7-24.4] | 4 (5.4) [2.1-13.6] | 8 (13.4) [4.2-35.5] | 11 (8.1) [4.3-14.8] |
| Always | 19 (9.2) [4.8-16.9] | 4 (4.5) [1.8-11.3] | 15 (12.8) [5.9-25.7] | 1 (9.8) [1.3-46.7] | 6 (8.7) [2.5-26.1] | 7 (14.7) [5.1-35.5] | 4 (5.4) [1.6-16.6] | 10 (16.4) [6.1-37.5] | 8 (6.0) [2.8-12.5] |
| Reason | | | | | | | | | |
| Lack of faith in police | 19 (15.2) [8.2-26.7] | 6 (14.2) [3.5-43.4] | 13 (15.7) [8.0-28.6] | 3 (38.0) [8.3-80.6] | 10 (19.2) [8.1-39.2] | 1 (0.3) [0.0-2.1] | 6 (17.5) [5.3-44.8] | 1 (2.2) [0.4-12.2] | 18 (22.5) [12.0-38.0] |
| Required for job | 6 (5.5) [1.8-15.1] | 1 (1.1) [0.2-8.1] | 6 (7.6) [2.4-21.4] | 0 | 3 (5.5) [1.5-18.1] | 4 (12.4) [2.2-46.7] | 1 (0.9) [0.1-6.3] | 3 (7.3) [1.0-37.8] | 4 (4.4) [1.6-12.0] |
| Expression of freedom | 12 (9.9) [5.6-16.8] | 2 (6.0) [1.7-19.5] | 10 (11.7) [6.2-21.1] | 1 (18.6) [2.3-68.9] | 4 (7.6) [2.8-18.8] | 3 (11.3) [3.5-30.6] | 4 (10.2) [4.0-23.8] | 2 (5.4) [1.6-17.0] | 10 (12.4) [6.5-22.1] |
| Self-protection | 104 (84.9) [74.1-91.7] | 34 (85.9) [69.2-94.3] | 70 (84.5) [69.5-92.8] | 7 (100) | 42 (83.0) [61.9-93.6] | 25 (85.4) [53.9-96.7] | 30 (84.3) [69.7-92.6] | 36 (80.7) [54.1-93.7] | 69 (87.3) [77.9-93.0] |
| Protect others | 69 (56.3) [44.1-67.9] | 12 (30.5) [16.1-50.1] | 57 (68.7) [54.1-80.4] | 4 (53.2) [13.8-89.0] | 29 (58.3) [36.8-77.0] | 17 (58.5) [35.1-78.6] | 19 (52.5) [34.1-70.2] | 24 (53.7) [31.8-74.2] | 46 (57.8) [43.6-70.8] |
| Hunting or recreation | 34 (28.0) [18.6-39.9] | 9 (23.4) [9.4-47.3] | 25 (30.2) [18.9-44.6] | 0 | 15 (30.5) [15.1-52.1] | 3 (9.5) [3.4-23.5] | 16 (45.3) [27.7-64.2] | 10 (22.4) [9.3-44.9] | 25 (31.1) [19.6-45.6] |
| Location | | | | | | | | | |
| Vehicle | 102 (83.2) [72.9-90.2] | 34 (85.6) [69.9-93.8] | 68 (82.1) [68.0-90.8] | 7 (92.8) [57.8-99.2] | 39 (77.0) [54.9-90.2] | 25 (85.4) [69.7-93.7] | 32 (88.2) [74.5-95.0] | 36 (82.4) [59.6-93.7] | 66 (83.7) [71.8-91.1] |
| Walking on the street | 60 (49.0) [37.2-60.9] | 10 (26.1) [12.4-46.7] | 50 (60.0) [45.2-73.2] | 5 (71.2) [27.8-94.1] | 24 (46.8) [27.3-67.3] | 17 (57.3) [34.1-77.7] | 15 (40.8) [24.5-59.5] | 24 (55.0) [33.0-75.2] | 36 (45.6) [32.3-59.6] |
| Retail stores | 56 (45.4) [33.8-57.5] | 11 (28.4) [14.2-48.7] | 45 (53.5) [39.1-67.4] | 5 (74.6) [32.6-94,7] | 19 (38.2) [20.8-59.3] | 20 (67.7) [45.8-83.8] | 11 (31.2) [17.4-49.5] | 20 (45.4) [25.2-67.2] | 36 (45.4) [32.1-59.4] |
| Restaurants | 49 (39.2) [28.3-51.4] | 10 (24.6) [11.6-44.9] | 39 (46.2) [32.3-60.7] | 4 (56.0) [16.5-89.1] | 18 (35.8) [19.0-57.1] | 16 (54.3) [31.6-75.3] | 10 (28.2) [15.2-46.3] | 21 (47.0) [26.6-68.6] | 28 (34.9) [23.1-48.8] |
| Places of worship | 32 (25.5) [16.6-37.2] | 6 (14.5) [6.1-31.0] | 26 (30.8) [19.0-45.8] | 1 (19.4) [2.4-70.0] | 15 (29.3) [14.1-51.2] | 7 (24.6) [10.5-47.6] | 8 (22.3) [10.5-41.1] | 16 (35.5) [17.8-58.5] | 16 (20.0) [11.5-32.4] |
| Parks | 46 (37.2) [26.4-49.4] | 9 (22.9) [10.4-43.2] | 37 (44.0) [30.2-58.7] | 5 (74.6) [32.6-94.7] | 19 (38.2) [20.7-59.4] | 12 (41.4) [21.0-65.2] | 9 (24.9) [12.5-43.3] | 19 (43.6) [23.8-65.8] | 27 (33.6) [21.9-47.6] |
| Others' homes | 29 (24.1) [15.3-35.8] | 5 (13.6) [5.5-30.0] | 24 (29.1) [17.5-44.3] | 1 (19.4) [2.4-70.0] | 16 (31.6) [15.8-53.3] | 5 (17.3) [5.8-41.5] | 7 (20.2) [9.2-38.6] | 15 (34.6) [17.1-57.5] | 14 (18.3) [10.1-30.7] |
| **Firearm violence exposure** | | | | | | | | | |

© 2024 Anestis MD et al. *JAMA Network Open.*

| Characteristic | American Indian or Alaska Native adults, No. (%) [95% CI] | | | | | | | | |
|---|---|---|---|---|---|---|---|---|---|
| | Total | Sex | | Age, y | | | | Metropolitan area status | |
| | | Female | Male | 18-29 | 30-44 | 45-59 | ≥60 | Nonmetropolitan | Metropolitan |
| Threatened with firearm | 158 (30.2) [24.8-36.3] | 63 (22.8) [16.2-31.1] | 95 (38.6) [30.1-48.0] | 16 (19.4) [7.8-40.6] | 62 (32.4) [23.2-43.1] | 50 (39.9) [28.4-52.6] | 31 (24.5) [17.2-33.7] | 42 (32.2) [20.9-46.0] | 117 (29.6) [23.5-36.4] |
| Shot | 33 (6.2) [3.9-9.8] | 11 (3.9) [1.5-9.9] | 22 (8.8) [5.2-14.4] | 2 (2.0) [0.4-10.2] | 14 (7.4) [3.6-14.6] | 11 (8.6) [3.2-20.8] | 6 (4.7) [2.5-8.8] | 2 (1.2) [0.4-3.3] | 31 (7.9) [4.8-12.6] |
| Friend or family member shot | 201 (38.4) [32.5-44.5] | 92 (33.2) [25.9-41.4] | 109 (44.2) [35.2-53.5] | 14 (16.3) [7.7-31.3] | 86 (44.8) [34.3-55.7] | 55 (43.8) [32.2-56.1] | 47 (37.7) [32.2-56.1] | 43 (33.2) [22.2-46.3] | 158 (40.1) [33.5-47.1] |
| Shooting in neighborhood | 147 (27.9) [22.9-33.5] | 83 (29.8) [23.1-37.5] | 64 (25.7) [18.7-34.3] | 25 (29.7) [16.7-47.3] | 52 (27.2) [18.9-37.4] | 39 (31.6) [21.9-43.4] | 30 (24.0) [16.6-33.3] | 30 (22.9) [14.4-34.5] | 117 (29.5) [23.7-36.1] |

[a]Rarely, 1% to 25% of the time; occasionally, 26% to 50%; often, 51% to 75%; almost always, 76% to 99%.

© 2024 Anestis MD et al. *JAMA Network Open.*

**eTable 4.** Regional and Political Group Differences in Firearm Access, Behaviors, and Exposure Among American Indian or Alaska Native Adults

| Characteristic | American Indian or Alaska Native adults, No. (%) [95% CI] | | | | | | | | |
|---|---|---|---|---|---|---|---|---|---|
| | Region | | | | Political beliefs | | | | |
| | Northeast | Midwest | South | West | Highly conservative | Somewhat conservative | Moderate | Somewhat liberal | Highly liberal |
| **Firearm in or around home** | | | | | | | | | |
| Any | 12 (27.8) [12.6-50.7] | 39 (48.7) [34.6-63.1] | 113 (59.1) [49.5-68.0] | 75 (35.6) [26.6-45.7] | 42 (72.6) [55.2-85.1] | 66 (54.7) [40.3-68.4] | 82 (38.1) [29.8-47.2] | 32 (46.5) [31.8-61.8] | 15 (28.7) [16.4-45.1] |
| Handguns, No. | | | | | | | | | |
| 0 | 2 (16.4) [3.6-51.0] | 3 (11.6) [4.6-26.1] | 12 (12.5) [6.8-22.0] | 12 (16.7) [7.4-33.4] | 1 (4.2) [1.1-15.2] | 9 (15.4) [7.4-29.4] | 8 (11.7) [4.3-28.0] | 7 (22.4) [9.3-44.8] | 3 (18.5) [6.8-41.4] |
| 1 | 2 (16.1) [2.1-63.0] | 12 (40.6) [22.4-61.9] | 40 (40.7) [29.4-53.0] | 26 (36.8) [23.5-52.5] | 9 (28.6) [12.9-51.9] | 21 (36.6) [21.8-54.4] | 28 (38.1) [25.8-52.3] | 14 (43.3) [23.8-65.1] | 8 (52.0) [28.4-74.8] |
| 2-4 | 8 (67.5) [29.1-91.3] | 12 (39.2) [19.1-63.8] | 38 (38.6) [27.2-51.5] | 27 (38.1) [24.4-54.0] | 17 (55.2) [32.4-76.0] | 22 (37.5) [22.2-55.7] | 30 (41.9) [28.6-56.5] | 10 (32.5) [14.5-57.8] | 5 (29.5) [11.8-56.5] |
| ≥5 | 0 | 3 (8.5) [1.5-33.1] | 8 (8.2) [4.2-15.4] | 6 (8.4) [2.1-27.6] | 4 (12.0) [3.6-32.8] | 6 (10.6) [2.9-32.2] | 6 (8.3) [3.6-18.0] | 1 (1.8) [0.4-7.4] | 0 |
| Shotguns, No. | | | | | | | | | |
| 0 | 7 (62.7) [21.4-91.2] | 13 (44.8) [25.3-66.0] | 37 (38.1) [27.5-49.9] | 24 (34.1) [21.7-49.1] | 13 (44.0) [23.2-67.2] | 20 (34.4) [20.6-51.3] | 27 (37.3) [25.4-50.9] | 12 (37.8) [20.2-59.3] | 9 (55.3) [30.8-77.6] |
| 1 | 1 (8.6) [2.1-29.6] | 6 (19.2) [8.6-37.8] | 34 (35.5) [24.2-48.6] | 32 (46.0) [30.7-62.1] | 6 (18.6) [7.7-38.4] | 29 (50.4) [33.3-67.3] | 21 (28.3) [16.4-44.2] | 15 (48.5) [27.5-70.1] | 3 (18.2) [5.8-44.5] |
| 2-4 | 3 (28.7) [4.5-77.6] | 9 (29.3) [11.1-57.9] | 21 (21.5) [12.9-33.5] | 14 (19.9) [11.1-33.0] | 9 (31.5) [12.6-59.5] | 8 (14.3) [6.4-29.0] | 21 (28.3) [16.8-43.5] | 4 (13.7) [4.2-36.4] | 4 (26.5) [9.6-55.1] |
| ≥5 | 0 | 2 (6.7) [0.9-35.2] | 5 (5.0) [2.0-11.9] | 0 | 2 (6.0) [0.8-32.7] | 1 (1.0) [0.1-6.7] | 4 (6.1) [2.2-16.0] | 0 | 0 |
| Rifles, No. | | | | | | | | | |
| 0 | 2 (20.8) [5.4-55.0] | 14 (46.4) [25.5-68.7] | 38 (39.1) [28.2-51.2] | 18 (26.2) [15.4-40.8] | 11 (37.5) [17.0-63.9] | 18 (31.3) [18.5-47.7] | 22 (30.4) [19.5-44.2] | 12 (36.7) [18.7-59.4] | 9 (60.3) [35.2-81.0] |
| 1 | 3 (28.0) [7.1-66.5] | 8 (26.1) [13.2-45.1] | 29 (29.5) [19.3-42.3] | 22 (31.1) [18.1-47.9] | 3 (11.1) [3.2-32.1] | 17 (29.0) [15.4-47.7] | 26 (36.2) [23.4-51.3] | 12 (37.8) [20.0-59.7] | 3 (16.3) [4.2-46.5] |
| 2-4 | 3 (22.5) [3.2-71.8] | 4 (13.8) [5.2-31.9] | 20 (20.2) [12.0-32.0] | 25 (36.0) [22.1-52.6] | 12 (38.6) [19.6-61.9] | 18 (31.6) [16.7-51.5] | 11 (14.6) [8.2-24.7] | 8 (24.5) [8.8-52.0] | 3 (21.0) [7.6-46.2] |
| ≥5 | 3 (28.7) [4.5-77.6] | 4 (13.7) [4.4-35.4] | 11 (11.2) [5.4-21.6] | 5 (6.8) [2.5-17.1] | 4 (12.7) [4.0-33.9] | 5 (8.1) [3.1-19.5] | 14 (18.8) [9.2-34.6] | 1 (1.0) [0.1-7.2] | 1 (2.4) [0.3-15.7] |
| **Primary firearm reason** | | | | | | | | | |
| Gift or inheritance | 0 | 4 (14.9) [6.3-31.3] | 7 (6.9) [3.6-12.7] | 3 (5.1) [1.9-13.0] | 2 (8.3) [2.2-26.3] | 3 (5.2) [2.1-12.1] | 1 (2.0) [0.6-6.5] | 4 (13.0) [5.5-27.7] | 4 (24.3) [9.3-50.1] |

© 2024 Anestis MD et al. *JAMA Network Open.*

| Characteristic | American Indian or Alaska Native adults, No. (%) [95% CI] | | | | | | | | |
|---|---|---|---|---|---|---|---|---|---|
| | Region | | | | Political beliefs | | | | |
| | Northeast | Midwest | South | West | Highly conservative | Somewhat conservative | Moderate | Somewhat liberal | Highly liberal |
| Safety at home | 9 (89.8) [61.1-98.0] | 9 (30.9) [15.7-51.7] | 56 (58.2) [46.0-69.4] | 41 (62.4) [46.0-76.3] | 16 (54.1) [30.5-76.0] | 39 (73.5) [56.2-85.8] | 43 (59.6) [45.0-72.7] | 14 (44.2) [23.6-67.1] | 3 (21.1) [7.2-47.9] |
| Safety away from home | 1 (5.6) [0.7-34.5] | 7 (23.4) [6.8-56.3] | 6 (6.2) [2.6-14.1] | 1 (1.7) [0.2-11.1] | 6 (18.6) [4.0-55.5] | 1 (2.4) [0.4-12.2] | 8 (10.8) [4.9-22.2] | 0 | 0 |
| Hunting | 0 | 2 (5.3) [1.1-22.8] | 6 (6.6) [3.2-13.1] | 8 (11.9) [3.8-31.3] | 2 (6.2) [1.4-22.9] | 2 (3.5) [0.7-16.3] | 10 (14.2) [5.9-30.2] | 2 (6.0) [1.5-21.3] | 0 |
| Other recreation | 0 | 1 (0.9) [0.1-6.3] | 1 (1.2) [0.3-5.5] | 3 (4.1) [1.3-11.8] | 1 (5.0) [1.3-17.3] | 1 (1.7) [0.2-11.7] | 0 | 1 (0.8) [0.1-5.9] | 1 (9.6) [2.0-35.5] |
| Belongs to someone else | 1 (4.7) [0.5-30.4] | 7 (23.7) [10.7-44.8] | 13 (12.9) [6.9-23.0] | 6 (9.3) [3.9-20.9] | 1 (4.6) [0.6-27.0] | 4 (7.6) [2.8-18.9] | 6 (8.7) [3.6-19.4] | 7 (22.9) [9.5-45.5] | 7 (45.0) [22.1-70.3] |
| **Firearm storage[a]** | | | | | | | | | |
| ≥1 Loaded | | | | | | | | | |
| Never | 5 (51.7) [14.9-86.8] | 10 (35.4) [19.8-54.8] | 38 (41.9) [30.3-54.5] | 32 (47.4) [31.4-63.9] | 11 (45.2) [24.5-67.7] | 15 (28.1) [16.0-44.6] | 30 (43.0) [29.0-58.3] | 18 (59.3) [35.6-79.4] | 10 (67.1) [40.8-85.8] |
| Rarely | 0 | 4 (15.9) [5.8-36.5] | 4 (4.3) [1.9-9.3] | 3 (4.1) [1.4-11.2] | 1 (5.5) [0.8-30.9] | 2 (4.0) [1.3-11.7] | 2 (3.6) [1.3-9.8] | 3 (10.4) [3.7-26.0] | 1 (8.9) [2.1-31.1] |
| Occasionally | 1 (4.7) [0.5-30.4] | 1 (1.2) [0.2-8.4] | 8 (8.7) [3.2-21.4] | 5 (8.5) [2.7-23.6] | 1 (2.6) [0.4-16.9] | 6 (11.2) [3.2-32.8] | 7 (10.0) [3.8-23.6] | 1 (2.8) [0.4-15.2] | 0 |
| Often | 0 | 0 | 3 (3.1) [0.8-11.4] | 1 (0.5) [0.1-3.6] | 1 (0.3) [0.0-2.5] | 2 (2.9) [0.4-18.5] | 1 (1.8) [0.2-11.7] | 1 (1.1) [0.1-7.6] | 0 |
| Almost always | 1 (2.1) [0.2-15.6] | 0 | 4 (4.5) [1.8-10.5] | 6 (7.4) [2.1-22.6] | 2 (8.6) [2.6-25.0] | 3 (2.7) [0.4-17.1] | 5 (8.1) [2.8-21.1] | 0 | 0 |
| Always | 4 (41.6) [9.7-82.5] | 11 (47.5) [28.4-67.4] | 40 (37.5) [26.1-50.6] | 20 (32.1) [18.0-50.5] | 10 (37.6) [19.5-60.0] | 27 (51.0) [33.1-68.7] | 26 (33.6) [21.4-48.4] | 10 (26.4) [9.5-55.2] | 3 (24.0) [8.6-51.5] |
| ≥1 With locking device | | | | | | | | | |
| Never | 6 (61.2) [20.6-90.6] | 11 (49.3) [30.1-68.7] | 43 (43.4) [31.5-56.2] | 18 (28.4) [16.9-43.7] | 10 (41.1) [22.0-63.3] | 22 (42.0) [25.2-60.9] | 21 (29.4) [18.0-44.2] | 18 (54.2) [31.5-75.3] | 7 (49.7) [25.4-74.1] |
| Rarely | 0 | 1 (6.0) [0.8-32.6] | 4 (4.4) [1.9-10.1] | 5 (7.3) [2.5-19.6] | 4 (14.6) [4.5-38.0] | 0 | 4 (6.6) [2.5-16.5] | 0 | 2 (15.5) [4.2-43.1] |
| Occasionally | 0 | 1 (2.9) [0.7-11.4] | 2 (2.3) [0.7-7.0] | 4 (6.2) [1.5-22.4] | 1 (3.5) [0.8-14.5] | 2 (3.7) [1.1-11.4] | 4 (5.7) [1.3-21.5] | 0 | 0 |
| Often | 0 | 0 | 1 (1.3) [0.4-3.5] | 6 (9.2) [2.4-30.0] | 0 | 1 (0.4) [0.1-2.8] | 1 (1.6) [0.5-5.1] | 5 (18.4) [4.5-52.0] | 1 (2.5) [0.3-16.7] |
| Almost always | 0 | 1 (2.4) [0.5-9.5] | 3 (3.4) [1.5-7.5] | 10 (15.1) [5.4-35.6] | 1 (1.4) [0.2-9.8] | 7 (13.0) [3.7-36.5] | 5 (7.2) [2.2-20.8] | 1 (1.9) [0.4-7.7] | 1 (5.3) [0.7-30.3] |

© 2024 Anestis MD et al. *JAMA Network Open.*

| Characteristic | American Indian or Alaska Native adults, No. (%) [95% CI] | | | | | | | | |
|---|---|---|---|---|---|---|---|---|---|
| | Region | | | | Political beliefs | | | | |
| | Northeast | Midwest | South | West | Highly conservative | Somewhat conservative | Moderate | Somewhat liberal | Highly liberal |
| Always | 4 (38.8) [9.4-79.4] | 11 (39.4) [22.2-59.7] | 41 (45.3) [33.2-57.9] | 21 (33.7) [20.1-50.5] | 10 (39.4) [19.8-63.1] | 22 (40.9) [24.8-59.3] | 34 (49.5) [35.0-64.1] | 8 (25.6) [11.4-47.8] | 4 (27.0) [10.1-55.1] |
| ≥1 In locked location | | | | | | | | | |
| Never | 3 (24.7) [4.9-67.7] | 7 (31.7) [17.3-50.7] | 26 (26.2) [16.7-38.7] | 12 (16.3) [8.6-28.9] | 6 (24.7) [10.8-47.1] | 12 (21.2) [9.8-40.1] | 10 (14.8) [8.0-25.9] | 13 (35.4) [18.2-57.4] | 6 (44.3) [21.5-69.8] |
| Rarely | 1 (4.5) [0.5-29.6] | 2 (8.8) [2.0-31.3] | 7 (7.2) [2.2-20.6] | 1 (0.5) [0.1-3.6] | 1 (5.5) [0.8-30.9] | 5 (9.1) [2.0-32.6] | 2 (3.5) [1.1-10.6] | 1 (1.1) [0.1-7.7] | 1 (3.6) [0.5-22.3] |
| Occasionally | 0 | 1 (1.0) [0.1-7.1] | 3 (3.7) [0.8-15.2] | 1 (1.9) [0.4-7.7] | 0 | 1 (1.9) [0.3-12.5] | 1 (0.7) [0.1-4.8] | 3 (11.4) [2.6-37.9] | 0 |
| Often | 0 | 1 (1.3) [0.2-9.3] | 3 (1.6) [0.6-4.4] | 3 (4.0) [1.2-12.5] | 1 (1.3) [0.2-9.3] | 1 (0.6) [0.1-4.2] | 3 (1.2) [0.3-4.9] | 2 (5.8) [1.7-18.0] | 1 (9.1) [1.3-43.7] |
| Almost always | 1 (5.1) [0.6-32.5] | 1 (1.4) [0.2-9.4] | 6 (6.6) [3.0-13.8] | 9 (13.7) [5.5-30.4] | 1 (2.8) [0.4-18.1] | 2 (4.3) [0.9-18.6] | 12 (17.9) [8.9-32.6] | 1 (2.2) [0.5-8.8] | 0 |
| Always | 7 (65.7) [26.1-91.2] | 18 (55.8) [36.2-73.7] | 50 (54.7) [42.1-66.8] | 42 (63.6) [47.5-77.1] | 21 (65.6) [43.2-82.7] | 34 (62.8) [43.6-78.7] | 41 (62.0) [47.4-74.7] | 13 (44.3) [23.2-67.6] | 7 (43.1) [20.5-69.0] |
| ≥1 In vehicle unlocked | | | | | | | | | |
| Never | 8 (74.9) [24.5-96.5] | 20 (80.3) [58.4-92.2] | 65 (69.6) [56.4-80.1] | 46 (67.0) [48.1-81.6] | 11 (45.4) [25.1-67.3] | 39 (70.1) [48.3-85.5] | 52 (73.1) [58.1-84.1] | 23 (76.4) [45.7-92.6] | 13 (90.0) [53.6-98.6] |
| Rarely | 0 | 2 (7.4) [1.4-30.5] | 9 (9.6) [4.6-19.2] | 10 (15.7) [6.1-35.0] | 6 (25.5) [10.7-49.6] | 6 (11.0) [2.8-34.5] | 8 (11.9) [5.0-25.9] | 1 (1.1) [0.1-7.6] | 0 |
| Occasionally | 0 | 0 | 2 (2.2) [0.7-6.8] | 5 (7.8) [1.5-31.1] | 1 (1.4) [0.2-9.8] | 2 (3.6) [0.9-13.9] | 1 (1.0) [0.2-4.4] | 4 (13.6) [2.0-54.3] | 0 |
| Often | 0 | 1 (4.7) [0.6-27.1] | 2 (2.4) [0.8-7.5] | 1 (0.9) [0.1-6.1] | 1 (3.4) [0.5-21.2] | 0 | 2 (3.4) [1.0-10.8] | 1 (2.5) [0.3-16.5] | 0 |
| Almost always | 0 | 6 (5.2) [0.7-29.4] | 3 (3.4) [0.9-11.7] | 0 | 0 | 1 (2.3) [0.3-14.9] | 1 (2.0) [0.4-8.1] | 2 (6.1) [0.8-33.0] | 0 |
| Always | 3 (25.1) [3.5-75.5] | 1 (2.4) [0.3-15.4] | 12 (12.7) [5.4-27.2] | 5 (8.6) [2.6-24.8] | 6 (24.3) [9.4-49.8] | 7 (13.0) [3.4-38.8] | 6 (8.7) [2.9-23.3] | 1 (0.3) [0.0-2.1] | 1 (10.0) [1.4-46.4] |
| **Firearm carrying** | | | | | | | | | |
| Frequency | | | | | | | | | |
| Never | 6 (54.9) [16.2-88.5] | 13 (44.3) [24.9-65.7] | 32 (33.2) [23.2-44.9] | 29 (43.4) [28.5-59.6] | 8 (26.9) [11.0-52.3] | 15 (26.6) [15.1-42.5] | 22 (30.7) [19.0-45.4] | 22 (69.9) [44.0-87.3] | 12 (84.4) [57.3-95.6] |
| Rarely | 1 (8.6) [1.6-35.2] | 4 (13.7) [5.3-31.0] | 22 (22.5) [13.7-34.6] | 22 (32.7) [18.7-50.6] | 4 (13.1) [5.3-28.9] | 18 (32.5) [17.6-52.0] | 19 (25.9) [15.3-40.3] | 6 (18.9) [5.2-49.8] | 2 (15.6) [4.4-42.6] |

© 2024 Anestis MD et al. *JAMA Network Open.*

| Characteristic | American Indian or Alaska Native adults, No. (%) [95% CI] | | | | | | | | |
|---|---|---|---|---|---|---|---|---|---|
| | Region | | | | Political beliefs | | | | |
| | Northeast | Midwest | South | West | Highly conservative | Somewhat conservative | Moderate | Somewhat liberal | Highly liberal |
| Sometimes | 3 (32.0) [5.0-80.8] | 3 (11.6) [4.0-29.0] | 8 (7.9) [3.6-16.4] | 2 (3.0) [0.7-11.7] | 2 (8.3) [2.2-26.3] | 4 (7.5) [2.5-20.7] | 7 (10.0) [3.7-24.3] | 3 (8.0) [1.8-29.9] | 0 |
| Frequently | 0 | 1 (4.3) [0.7-21.0] | 14 (14.0) [7.3-25.0] | 5 (8.1) [2.6-22.6] | 3 (9.1) [2.8-25.9] | 4 (7.1) [1.9-23.1] | 3 (18.1) [9.2-32.7] | 1 (2.1) [0.3-14.1] | 0 |
| Almost always | 1 (4.5) [0.5-29.6] | 8 (26.1) [8.6-56.9] | 5 (5.5) [2.2-13.1] | 6 (9.4) [3.5-22.8] | 10 (35.2) [14.9-62.8] | 4 (7.0) [2.0-21.7] | 5 (7.6) [3.3-16.5] | 0 | 0 |
| Always | 0 | 0 | 16 (16.9) [8.6-30.6] | 2 (3.4) [0.6-16.6] | 2 (7.4) [1.4-30.6] | 11 (19.4) [7.5-41.6] | 6 (7.7) [3.0-18.7] | 1 (1.0) [0.1-7.1] | 0 |
| Reason | | | | | | | | | |
| Lack of faith in police | 0 | 1 (6.7) [1.3-28.5] | 7 (11.3) [4.8-24.5] | 10 (27.6) [11.0-53.9] | 1 (4.2) [1.0-16.0] | 5 (12.9) [2.8-42.9] | 12 (24.9) [12.5-43.5] | 1 (3.3) [0.4-24.2] | 0 |
| Required for job | 1 (10.0) [0.9-58.7] | 0 | 5 (7.4) [1.9-25.1] | 2 (4.0) [0.5-24.4] | 3 (14.1) [4.2-38.0] | 3 (8.1) [1.1-40.4] | 1 (0.9) [0.1-6.5] | 0 | 0 |
| Expression of freedom | 0 | 4 (22.2) [7.1-51.4] | 5 (7.6) [3.2-17.0] | 4 (9.6) [3.2-25.4] | 5 (24.5) [9.7-49.5] | 1 (1.1) [0.1-7.7] | 6 (11.6) [5.0-24.6] | 1 (7.0) [0.8-41.6] | 0 |
| Self-protection | 4 (93.3) [52.8] | 14 (83.6) [54.2-95.7] | 53 (82.1) [63.6-92.3] | 34 (89.4) [74.9-96.0] | 19 (88.7) [64.8-97.1] | 31 (76.9) [50.2-91.6] | 44 (89.2) [77.1-95.3] | 8 (84.3) [47.7-96.9] | 2 (100) |
| Protect others | 4 (93.3) [52.8-99.4] | 8 (50.3) [20.6-79.9] | 35 (53.8) [38.1-68.8] | 22 (58.9) [35.8-78.6] | 13 (57.4) [28.3-82.1] | 19 (47.4) [26.5-69.2] | 30 (60.2) [42.8-75.3] | 7 (72.2) [34.2-92.8] | 1 (54.3) [8.2-94.1] |
| Hunting or recreation | 1 (19.1) [2.3-69.9] | 4 (23.5) [8.0-52.0] | 15 (23.2) [12.6-38.8] | 15 (39.4) [19.9-63.0] | 6 (28.3) [11.8-53.8] | 13 (33.3) [15.3-58.0] | 14 (27.3) [14.6-45.2] | 1 (15.7) [3.1-52.3] | 0 |
| Location | | | | | | | | | |
| Vehicle | 4 (90.0) [41.3-99.1] | 13 (76.3) [47.4-92.0] | 56 (85.8) [69.8-94.0] | 30 (81.0) [58.8-92.7] | 19 (86.3) [62.0-96.0] | 34 (83.9) [58.6-95.0] | 42 (83.7) [67.0-92.8] | 7 (77.8) [40.0-94.9] | 1 (55.8) [8.6-94.4] |
| Walking on the street | 1 (6.7) [0.6-47.2] | 6 (36.6) [14.2-66.8] | 36 (55.1) [39.5-69.7] | 18 (49.0) [27.8-70.6] | 10 (44.8) [20.8-71.6] | 22 (54.1) [31.9-74.8] | 25 (50.7) [33.8-67.4] | 3 (29.2) [6.0-72.7] | 1 (45.7) [5.9-91.8] |
| Retail stores | 4 (87.6) [35.2-98.9] | 4 (23.8) [7.6-54.2] | 34 (53.2) [37.6-68.1] | 14 (36.3) [18.6-58.7] | 12 (52.9) [25.8-78.4] | 19 (47.5) [26.5-69.5] | 24 (48.4) [31.7-65.4] | 1 (3.3) [0.4-24.2] | 1 (45.7) [5.9-91.8] |
| Restaurants | 1 (16.7) [2.1-65.3] | 3 (15.3) [4.2-42.7] | 35 (53.9) [38.3-68.8] | 10 (27.1) [12.1-50.1] | 10 (43.8) [20.2-70.6] | 19 (47.5) [26.5-69.5] | 19 (37.7) [22.6-55.6] | 1 (11.2) [1.9-44.7] | 0 |
| Places of worship | 1 (10.0) [0.9-58.7] | 2 (11.8) [2.7-39.1] | 20 (30.2) [17.6-46.8] | 10 (25.3) [10.8-48.7] | 6 (29.3) [12.0-55.7] | 14 (34.2) [16.3-58.1] | 11 (22.3) [11.1-39.7] | 1 (3.3) [0.4-24.2] | 0 |
| Parks | 1 (6.7) [0.6-47.2] | 2 (13.4) [3.2-41.7] | 31 (47.5) [32.2-63.3] | 13 (33.5) [16.6-56.0] | 5 (22.3) [7.8-49.2] | 18 (44.7) [24.1-67.2] | 21 (42.2) [26.3-59.9] | 1 (11.2) [1.9-44.7] | 1 (45.7) [5.9-91.8] |
| Others' homes | 0 | 1 (1.4) [0.2-10.5] | 21 (32.2) [19.1-48.8] | 9 (23.0) [9.5-46.1] | 4 (17.2) [5.5-42.5] | 12 (29.1) [12.5-54.0] | 13 (25.8) [13.5-43.6] | 2 (15.8) [3.1-52.4] | 0 |

© 2024 Anestis MD et al. *JAMA Network Open.*

| Characteristic | American Indian or Alaska Native adults, No. (%) [95% CI] | | | | | | | | |
|---|---|---|---|---|---|---|---|---|---|
| | Region | | | | Political beliefs | | | | |
| | Northeast | Midwest | South | West | Highly conservative | Somewhat conservative | Moderate | Somewhat liberal | Highly liberal |
| **Firearm violence exposure** | | | | | | | | | |
| Threatened with firearm | 16 (36.3) [16.0-63.0] | 17 (21.1) [12.8-32.6] | 64 (33.3) [24.9-42.9] | 63 (29.6) [20.9-40.1] | 21 (35.9) [20.4-55.1] | 37 (30.7) [19.0-45.6] | 63 (29.0) [21.2-38.3] | 28 (40.5) [26.0-57.0] | 10 (18.8) [9.5-33.7] |
| Shot | 1 (2.7) [0.4-17.3] | 3 (3.8) [1.3-10.7] | 15 (8.1) [3.9-16.0] | 13 (6.2) [3.0-12.4] | 9 (15.0) [4.8-38.5] | 3 (2.4) [0.8-6.6] | 17 (7.8) [4.3-13.7] | 3 (5.0) [1.3-18.1] | 1 (0.4) [0.0-2.7] |
| Friend or family member shot | 19 (42.8) [22.7-65.6] | 20 (25.1) [16.1-36.9] | 69 (36.0) [27.6-45.3] | 94 (44.6) [34.3-55.4] | 25 (43.5) [26.8-61.9] | 37 (30.4) [19.3-44.4] | 92 (42.4) [33.5-51.8] | 33 (48.3) [33.4-63.6] | 15 (27.1) [14.7-44.3] |
| Shooting in neighborhood | 10 (23.1) [10.6-43.2] | 21 (26.6) [16.5-40.1] | 57 (29.7) [21.7-39.2] | 59 (27.7) [19.8-37.3] | 18 (32.0) [16.9-52.1] | 28 (23.2) [14.1-35.6] | 60 (27.6) [20.3-36.3] | 24 (35.3) [22.5-50.7] | 16 (29.3) [16.0-47.5] |

[a]Rarely, 1% to 25% of the time; occasionally, 26% to 50%; often, 51% to 75%; almost always, 76% to 99%.

© 2024 Anestis MD et al. *JAMA Network Open.*
